# Liquid-Based Reconfigurable Antenna Technology: Recent Developments, Challenges and Future

**DOI:** 10.3390/s21030827

**Published:** 2021-01-26

**Authors:** Habshah Abu Bakar, Rosemizi Abd Rahim, Ping Jack Soh, Prayoot Akkaraekthalin

**Affiliations:** 1 Department of Electrical Engineering, Politeknik Sultan Abdul Halim Muadzam Shah, Jitra 06000, Kedah, Malaysia; abubakarhabshah@gmail.com; 2 Faculty of Electronic Engineering Technology, Pauh Putra Campus, Universiti Malaysia Perlis, Pauh 02600, Perlis, Malaysia; pjsoh@unimap.edu.my; 3 Advanced Communication Engineering (ACE) Centre of Excellence, Universiti Malaysia Perlis, Kangar 01000, Perlis, Malaysia; 4 Department of Electrical and Computer Engineering, Faculty of Engineering, King Mongkut’s University of Technology North Bangkok (KMUTNB), 1518 Pracharat 1 Rd., Wongsawang, Bangsue, Bangkok 10800, Thailand

**Keywords:** reconfigurable antennas, liquid antennas, liquid actuation techniques

## Abstract

Advances in reconfigurable liquid-based reconfigurable antennas are enabling new possibilities to fulfil the requirements of more advanced wireless communication systems. In this review, a comparative analysis of various state-of-the-art concepts and techniques for designing reconfigurable antennas using liquid is presented. First, the electrical properties of different liquids at room temperature commonly used in reconfigurable antennas are identified. This is followed by a discussion of various liquid actuation techniques in enabling high frequency reconfigurability. Next, the liquid-based reconfigurable antennas in literature used to achieve the different types of reconfiguration will be critically reviewed. These include frequency-, polarization-, radiation pattern-, and compound reconfigurability. The current concepts of liquid-based reconfigurable antennas can be classified broadly into three basic approaches: altering the physical (and electrical) dimensions of antennas using liquid; applying liquid-based sections as reactive loads; implementation of liquids as dielectric resonators. Each concept and their design approaches will be examined, outlining their benefits, limitations, and possible future improvements.

## 1. Introduction

Antennas are one of the critical components in wireless communication systems, which must be designed and deployed efficiently. This is aimed at effectively accommodating multiple wireless services simultaneously [1]. Traditionally, conventional antennas are designed for operation in a fixed frequency band, with consistent radiation pattern and polarization. However, with the advent of the fifth generation (5G) wireless communication systems and beyond and the additional antenna requirements that come with it, the choice of the antenna and its features is becoming increasingly crucial. One of the most appealing features for future antennas is the capability for it to reconfigure their performance in front-ends, to enable an all-spectrum communication and multiband operation [2]. Besides that, channel capacity enhancement using the multiple input multiple output (MIMO) principle must also be improved by accommodating multiple antennas in future user devices. While increasing the number of elements generally translates to capacity improvement, the implementation of such designs must not compromise antenna performance such as mutual coupling and total efficiency. These factors also limit the actual number of antenna elements that can be integrated into compact mobile terminals or base stations, especially when designing electrically large, sub-6 GHz MIMO systems, besides considerations of the overall product design and user experience [3]. Due to the potential increase in total power consumption in implementing massive MIMO relative to a sub-6 GHz active antenna, researchers started to explore tunable materials such as liquid crystals to increase antenna agility [4]. Liquid crystal phase shifters can be integrated with the phased arrays and can be made tunable by applying low voltages, thus improving energy efficiency. Besides that, 5G is also envisioned to be a combination of massive MIMO and small cell networks; which are expected to employ optimal and low-complexity beamforming [5]. Finally, the deployment of 5G networks with massive MIMO antennas in practice require considerations on aspects such as user density, antenna density, and data rate requirements to balance energy and spectral efficiencies [6]. 

Reconfigurable antennas offer the capability to change the different antenna characteristics to adapt the dynamic wireless communication systems [7]. Reconfigurability is defined as the ability of antennas to change their operational frequency, radiation pattern, polarization, and other properties. Conventional reconfigurable antennas typically employ techniques such as radio frequency (RF) switches such as PIN diodes [1,7] and varactors [8]. Besides that, microelectromechanical system (MEMS) switches, which offer high isolation and low insertion loss [9] are also becoming popular, whereas PIN diodes are ideal for fast switching [10] and varactor diodes provide continuous tuning ability [11]. Despite being achievable using RF switches, the reconfigurability feature in antennas can also be implemented using conductive fluids such as liquid metal (LM), ionized water, and dielectric fluids such as ethyl acetate and deionized (DI) water. The use of liquid enables the antennas to be more conveniently implemented on flexible materials and consequently, combined with other sensing functionalities for wireless communication [12]. The prospect of implementing such reconfigurable antenna designs is further accelerated with the recent introduction of liquid metals such as Galinstan and EGaIn [13]. These metallic liquids feature excellent electrical and thermal conductivity, nontoxicity, and unique physical properties. They have been implemented for frequency reconfigurability [14], polarization reconfigurability [15], radiation pattern reconfigurability [16], gain reconfigurability [17], and the combined reconfiguration of multiple parameters, or compound reconfigurability [18]. These nontoxic liquid metals can be actuated within an antenna enclosure by applying pressure using a syringe or pump [19] or via electrical actuation such as electrocapillary actuation (ECA), continuous electrowetting (CEW), and electrochemically controlled capillary (ECC) [20]. Recent implementations of liquid metal in reconfigurable antennas include the likes of monopoles [21], dipoles [18], helical [15], planar inverted-Fs [22], slot antennas [23], and Yagi-Uda antennas [24].

Past reviews have discussed the different aspects of liquid-based antennas. For instance, the research progress on the fabrication techniques for microfluidic electronics, with a focus on stretchable substrates, their issues, and future challenges was reported in [25]. Besides that, a review on methods of manipulating the interfacial tension of liquid metals using voltage for reconfigurable antennas was presented in [20]. The major challenges faced in fabricating gallium-based liquid metals was discussed in [26], whereas the review in [27] focused on presenting the unique properties and applications of gallium-based alloys. The development of antennas using conductive and dielectric liquids was presented in [28], whereas the use of different substrates and conductive fluids, and their fabrication techniques to achieve single reconfigurability was reviewed in [29]. Finally, the article in [11] reviewed the design techniques, advantages, and limitations of liquid-based antennas, specifically focused on dielectric liquids. Despite this literature, none of these reviews have focused on the detailed concepts, design approaches, and implementation of conductive and dielectric liquids into different types of single-parameter and compound reconfigurable antennas. 

To address this gap, this paper reviews recent progress on reconfigurable antennas using conductive and dielectric fluids, including their concepts, designs, and implementations. This article covers four underlying areas of focus. First, it is focused primarily on nontoxic gallium-based alloys, which exists in liquid form in (or when close to) room temperature. Besides that, their properties and available actuation techniques are presented in Section 2. The next focus is on analyzing liquid-based techniques which have been successfully used to achieve various types of antenna reconfigurability in Section 3 and Section 4. Finally, a future perspective of such mechanism, highlighting the life expectancy of these liquids concludes this article. 

## 2. Nontoxic Liquid Metals

Liquid metals near or below room temperature offer great potential in the development of innovative antenna technology for realizing stretchable antennas [30], flexible antennas [31], and reconfigurable antennas [19]. This is due to their capability in maintaining stable electrical functions despite the shape deformation caused by stretching and compression. Generally, there are five metals which can remain as liquid metals at room temperature due to their melting points: caesium (Cs), francium (Fr), gallium (Ga), mercury (Hg), and rubidium (Rb) [27]. The explosive reaction of Cs and Rb, radioactivity of Fr, and toxicity of Hg limit their practical applications to specialized areas only [27]. Gallium, on the other hand, can be combined with other metals to create the binary alloy, eutectic gallium indium (EGaIn) and ternary alloy gallium, indium, and stannum (Galinstan). Due to its nontoxic property, the application of Gallium-based alloys is growing steadily in reconfigurable antenna technology [32]. Besides that, Gallium-based liquid metals also feature unique chemical properties and excellent electrical conductivity [33]. Manipulation of the physical and chemical properties of Gallium-based alloys such as Galinstan and EGaIn potentially contributes to the development of flexible and reconfigurable antennas.

### 2.1. Properties of Nontoxic Liquid Metal

Galinstan is an odorless and silvery liquid which exists at room temperature, composed of metal components gallium (68.5%), indium (21.5%), and tin or stannum (10%). It features an excellent electrical and thermal conductivity and unique chemical properties. It has a low melting point of −19 °C, a boiling point of above 1300 °C, and thermal conductivity of 16.5 W/mK. Due to the uniqueness of its chemical properties and its low-toxicity, Galinstan is an ideal alternative for mercury, which was previously employed in miniaturized devices [34]. On the other hand, EGaIn is an electrically conductive liquid metal composed of 75.5% Gallium and 24.5% indium. Its physical properties are similar to Galinstan, as summarized in Table 1, featuring a higher melting point and boiling point compared to Galinstan, at about 15.5 and 2000 °C.

### 2.2. Liquid Metal Actuation in Reconfigurable Antenna

**Figure 1:**
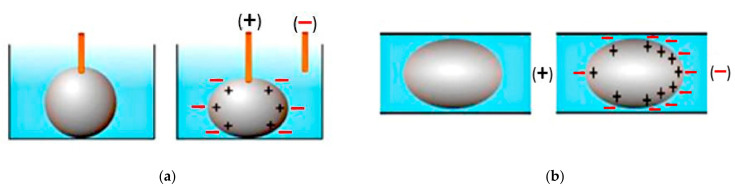
Illustrative comparison between (**a**) electrocapillary actuation (ECA) and (**b**) continuous electrowetting (CEW) [20].

**Figure 2:**
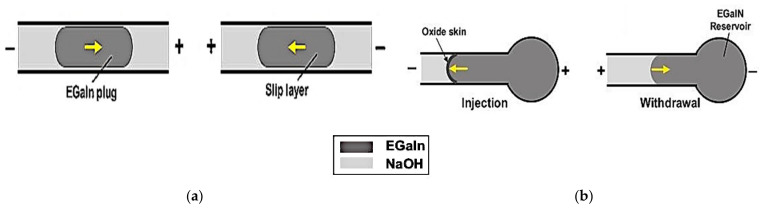
Illustration of the mechanism of (**a**) continuous electrowetting (CEW) and (**b**) electrochemically controlled capillary (ECC) [37].

Conventionally, liquid metal can be actuated using manual pneumatic actuation, either using the simplest form of a syringe [35], or by pneumatic actuation [36] using a pump or micropump [14]. Besides that, electronic actuation techniques such as electrocapillary actuation (ECA), continuous electrowetting (CEW), and electrochemically controlled capillary (ECC) using voltage difference are also effective in controlling the interfacial or surface tension of liquid metal. The basic mechanism of ECA is the change of the surface tension of liquid metals using electrical potential charges (electrical bias) at the boundary between two fluids, i.e., liquid metal and electrolyte. This thus deforms the shape of liquid metal in response to voltage. On the other hand, CEW operates based on variation in the gradients of the surface tension of the liquid metal [20]. The continuous change in the liquid metal surface wetting properties is caused by the potential drop of the liquid metal in an electrolyte. This is caused by electrocapillary, thereby allowing the liquid metal to be actuated within the channel. Figure 1 illustrates the comparison between ECA and CEW [20]. The third ECC method works based on the decrease of interfacial tension of the liquid metal by adding strong acids or bases. This is to continuously remove or reduce the excessive oxide layer that prevents the direct contact between the liquid metal and its surroundings. In comparison to CEW, changes in ECC in moving the liquid metal in the reservoir towards the capillary is more significant in response to the bias voltage, as illustrated in Figure 2 [37].

In [19], two flexible tubing have been inserted into holes to serve as liquid metal inlet and outlet for the antenna structure. A manual liquid actuation has been implemented by inserting syringes to inject the liquid into microchannel. This antenna has showed the same performance despite its channel being repeatedly washed using Teflon and refilled with Galinstan. The fluid inlet and outlet also has been introduced in [36], where a syringe is used to generate the pressure-driven air bubble actuation. In [32], the applied air pressure using syringe is used to control the physical length of the antenna. This process, while remaining repeatable and reversible, results in less radio frequency losses than CEW actuation. However, such manual actuation method is limited in terms of switching speed.

The automatic actuation of liquid metal using a computer-controlled syringe pump has been proposed and implemented in [22]. This pump can infuse and withdraw liquid metal in the upper arm of planar inverted-F antenna, allowing it to control its electrical length. A vector network analyzer (VNA) and syringe pump are connected to the control computer, resulting in a bulky system. Conversely, such system can be more practical and compact if a micropump is used in their place. The use of such micropump has been demonstrated in [14], where the amount of liquid metal injected into the microfluidic channels is determined using a micropump activated using a microcontroller. It has been also demonstrated in [38] that the physical length of the antenna can be reconfigured using bidirectional micropump units. This is done by retracting a portion of the volume of liquid-metal to reside over the feed line of the microstrip. Besides that, this reconfiguration technique also depends on the ability of the antenna to form a continuous liquid-metal slug in microfluidic channel. A peristaltic pump has been used to actuate the Galinstan flow for the helical antenna in [39]. Using a Raspberry Pi computer, the peristaltic pump is controlled via an H-bridge circuit that can continually adjust the pump speed and direction. For applications requiring compact sizes, the size of the micropump unit might be of concern in practice.

In [40], liquid metal was actuated electrically using ECA to place the liquid metal in polyimide channels. The applied DC voltage will manipulate liquid metal surface tension, causing motion towards positive bias. However, the DC bias must be supplied continuously to avoid the liquid metal withdrawing back to its original position. To overcome this need, a metastable locking method that extends the ECA concept is employed. Metastable locking can be achieved by implementing notches in the fluidic channel. The liquid metal extends to fill a notch in the channel, producing a minimal localized surface energy and rests the liquid metal in the notch. A DC bias with reverse polarity is supplied for the purpose of releasing the liquid metal from that notch. The same method has also been implemented in [41]. 

**Figure 3:**
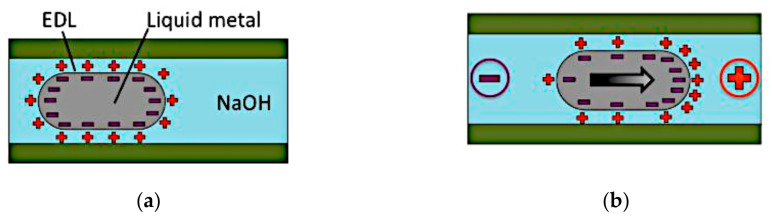
(**a**) Liquid metal in an electrolyte. (**b**) Potential gradient across the electrolyte [42].

The antenna presented in [42] implemented the CEW to induce motion of liquid metal based on its surface tension. When liquid metal is immersed in an electrolyte, the exchange of ions results in a net charge acquisition on the surface of the fluidic metal (Figure 3). This surface charge draws opposite charged ions from the adjacent electrolyte onto the polarizable interface, forming an electrical double layer (EDL). The motion of the liquid metal occurs due its attempt to minimize its surface energy by "wetting" it to the lower surface tension.

The CEW method also has been used in [17]. The gain of the reconfigurable antenna is controlled by applying a voltage bias through an electrolyte to induce the slug of Galinstan along the channel. This then tunes the length of stub and antenna gain. In [43], the CEW method is used to switch on and off the pixelated dipole by actuating the liquid metal to the top and bottom of the antenna reservoir. This technique controls the liquid metal tuning automatically, besides reducing the microscale device dimensions. Such concept paves the way for future submillimeter-dimensioned systems. 

A monopole antenna presented in [37] implemented the ECC method by supplying a DC potential between the EGaIn reservoir and electrolyte. The positive supply to the liquid metal increases the oxidation of the leading surface and reduces the surface tension of the metal fluid. This then moves the liquid metal towards the electrolyte in the capillary. Changing the voltage polarity will withdraw the liquid metal back into the reservoir, which also changes the shape and position of the antenna. On the other hand, ECC has been used in the cylindrical helical antenna to alter the length of liquid metal in the microfluidic channel in [44]. The EGaIn oxide layer which has been produced electrochemically increases the interfacial tension of liquid metal, thus driving the liquid metal to the channel. Such concept has also been applied in [18], where four independent DC bias voltages are applied to two pairs of dipole arms containing EGaIn liquid metal and electrolyte to control the lengths of each individual arm. The ECC method allows simultaneous injection or withdrawal of liquid metal from multiple capillaries. In addition to that, the use of strong acid and bases in the ECC method helps to continually remove excessive oxidation layers. In the following sections, examples of reconfigurable antennas using nontoxic liquid metal based on different design concepts and applied technology will be discussed.

## 3. Design Concepts of Nontoxic Liquid Metal Reconfigurable Antennas

The interest in reconfigurable antennas in the past few years has grown due to their capability of enabling tunability and switchability in antenna designs. While the implementation of RF switches such as PIN diodes [1] have increased the growth of such versatile antennas, the number and range of switching states increases with the increase of circuit complexity [37]. This motivated the choice of metal fluids, which changes the antenna characteristics using fluid flow as an alternative for antenna reconfiguration. In recent years, various unique and novel concepts of reconfigurable antennas using liquid metals have been developed. Several key aspects of antenna reconfiguration will be addressed in the following sections, with examples of antenna structures for different types of reconfigured parameters. The three main types of reconfigurable parameters are frequency reconfiguration, polarization reconfiguration, and other reconfigurations (compound, gain, phase shifting, and directivity reconfiguration).

### 3.1. Frequency Reconfigurable Antennas

Frequency reconfigurable antennas have become an important feature in modern communication systems. There are commonly two design approaches for achieving frequency reconfigurability, which are by altering the physical size of the antenna and reactive loading of liquid metal. Other possible techniques to achieve frequency reconfigurability will also be explained.

#### 3.1.1. Physical/Electrical Size Modification

**Figure 4:**
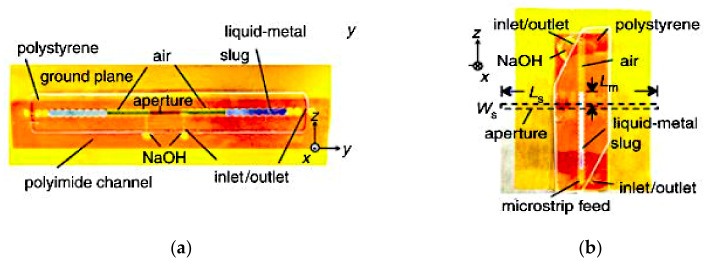
Fluidic slot antenna. (**a**) Liquid metal slugs in aperture. (**b**) Liquid metal slug in microstrip line [36].

Manipulating the length of the microstrip feed line and radiating aperture of a slot antenna to enable the frequency tuning has been studied in [36], as shown in Figure 4. 

Two fluidic channels are fabricated at either ends of the aperture and microstrip feed line. Air pressure difference is created between the two sides of the Galinstan slugs by pneumatically pumping the liquid using a syringe. Prior to that, a thin layer of sodium hydroxide (NaOH) solution is used to coat the Galinstan slugs in an air-filled hydrophobic channel. This is to remove residues of Galinstan and acts as actuation mechanism. Air bubbles will drive the slugs within the fluidic channels to adjust the length of the feed line and radiating aperture, thus achieving frequency reconfigurability from 1.42 to 1.84 GHz. This slot antenna achieved 26% of tunable bandwidth, with gains ranging from 4.1 to 4.8 dBi. This method, while remaining repeatable and reversible, minimized radio frequency losses compared to the CEW actuation. However, a longer actuation time (of about 1s) is required due to the use of syringe for pneumatic actuation.

**Figure 5:**
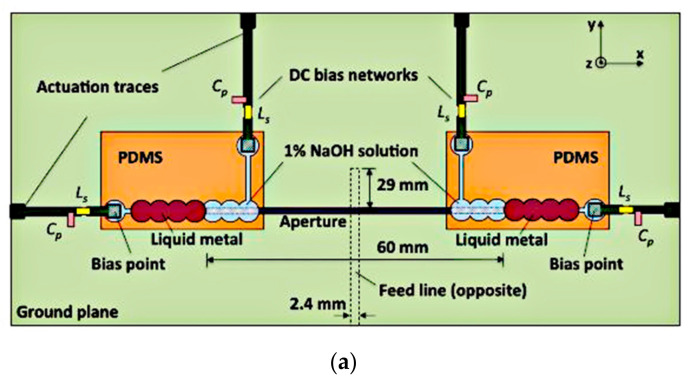

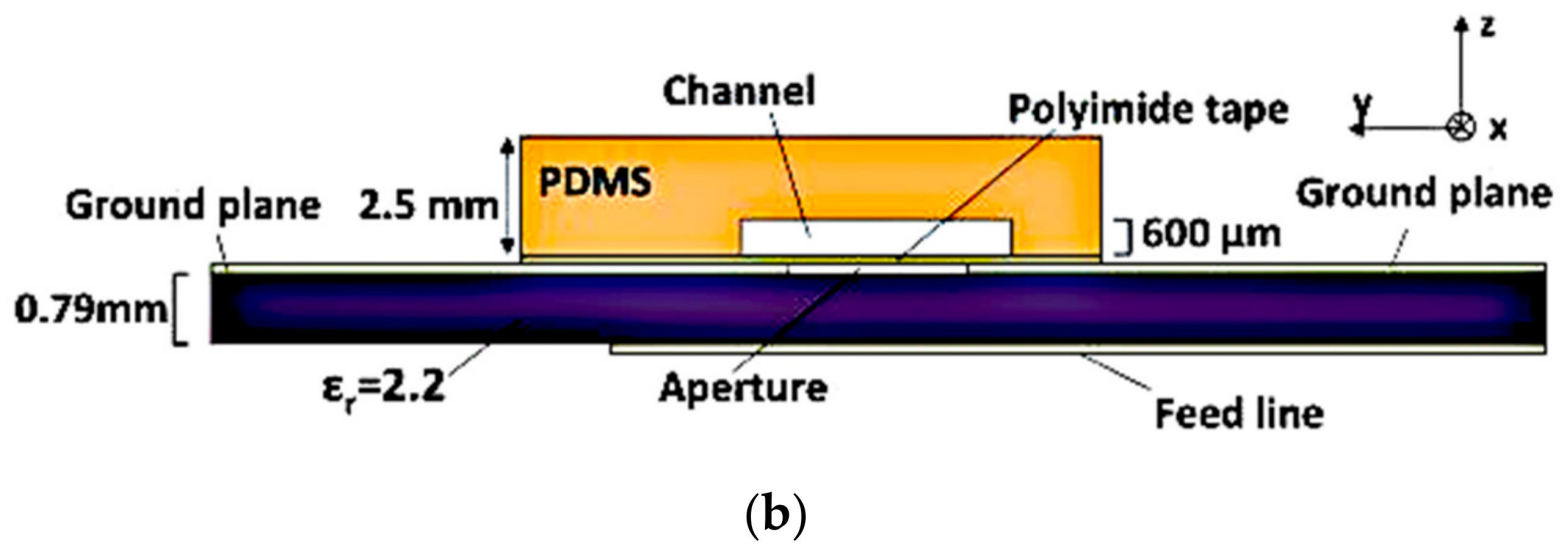
Frequency reconfigurable antenna. (**a**) Top view. (**b**) Side view [42].

Next, a frequency reconfigurable antenna using electrically actuated Galinstan is proposed in [42]. In this work, a fully sealed channel with integrated DC actuation network is introduced to improve the antenna robustness. Frequency reconfiguration is enabled using a Galinstan slug by varying the slot aperture, thus changing the resonant frequency of the antenna. An interlocking circular channel made using PDMS is fabricated at the two ends of the aperture and overlaps the slot line on each side. Polyimide tape is used to bond the PDMS fixture to the ground plane and to separate the liquid metal from copper, as illustrated in Figure 5. An 8Vpp, +3VDC square wave signal is applied to actuate the liquid metal towards the areas with lower surface tension. This antenna increased the operational frequency from 2.78 to 3.63 GHz and achieved 36% of total effective tuning bandwidth. 

**Figure 6:**
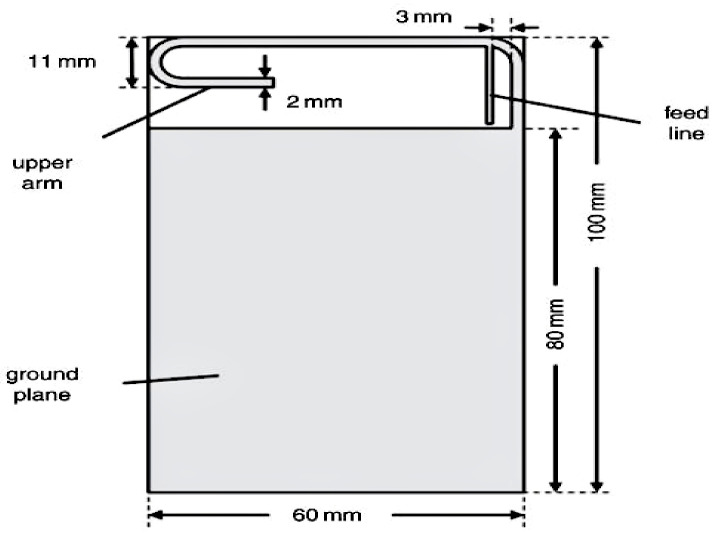
Planar inverted-F antenna (PIFA) [22].

Meanwhile, the upper arm of a planar inverted-F antenna (PIFA) with Galinstan liquid metal was presented in [22]. The Galinstan liquid is filled in a Teflon tube, as shown in Figure 6. To prevent the fluidic channel from being folded, each corner of the Teflon tube are rounded at a certain radius.

Experiments validated that the changes in the length of the Galinstan-filled portion changes the operating frequency. This is capable of automatically tuning the antenna back to the 720 MHz resonant frequency after an interference from the human hands placed near the antenna. However, the use of the syringe pump in this design is less practical.

**Figure 7:**
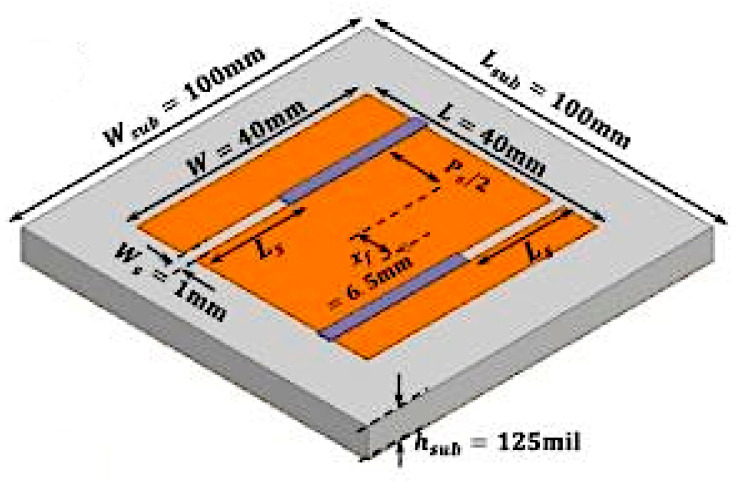
Patch antenna with two slotted patches [45].

Another example of frequency reconfigurable antenna with 3D printed microfluidic channel and composite tuning slots is presented in [45]. Two slots are inserted on a patch and the microfluidic channel is bounded on top of the slotted patch, as shown in Figure 7. By loading the liquid metal into the microfluidic channel, the length of liquid metal is altered, and thus produced 70% of frequency tuning bandwidth that ranged from 2 to 3.5 GHz.

**Figure 8:**
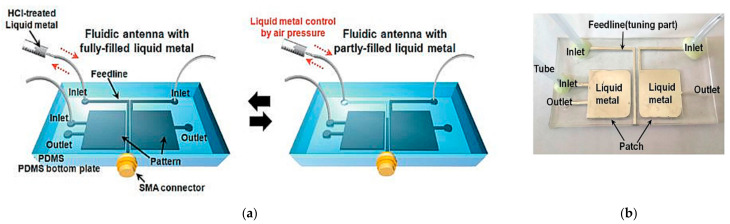
The proposed frequency tunable antenna. (**a**) Schematic antenna. (**b**) Fabricated antenna [32].

In [32], the frequency tunability of the fluidic antenna is enabled by changing the liquid metal feedline. In this design, a bottom and top plate are bonded together to form fluidic channels using plasma treatment. The feedline is designed in a “7”-shaped digital number, whereas a square-shaped and “6”-shaped pattern fluidic channel was fabricated on PDMS and filled with Galinstan, as shown in Figure 8. Air pressure is applied to control the physical length of the fluidic slug. The inlet and outlet ports are connected to the feedline and all fluidic channels to enable fluid injection. It has been demonstrated that by manipulating the length of the fluidic slug, the frequency range can be tuned from 2.2 to 9.3 GHz.

**Figure 9:**
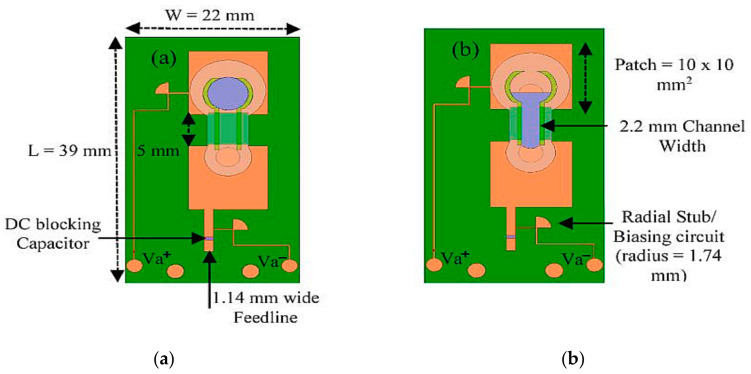
Dual patch antenna. (**a**) OFF configuration. (**b**) ON configuration [46].

Next, a tunable dual-band patch antenna was studied in [46]. This antenna was implemented using the 3D printing technique on a Polymethylmethacrylate (PMMA) substrate, and channels on a double patch antenna were fabricated using Fused Deposition Modeling (FDM). The PMMA channel consists of three layers, namely the foundation layer, the liquid metal channel, and the cover layer. A liquid metal channel with a width of 2.2 mm is used to connect (ON configuration) or disconnect (OFF configuration) the two patches, as shown in Figure 9. The external voltage is supplied to redrain and refill the liquid metal into the channels to achieve frequency reconfigurability. The two side channels are introduced to separate the boundaries of Galinstan and NaOH. This design includes a biasing circuit using a quarter wavelength inductive line and a DC blocking capacitor. The use of the capacitor leads to self-resonance, while the implementation of the 90° radial stub was aimed to obtain a wideband frequency. The simulated center frequency for the OFF and ON configurations ranges from 14.2 (with a bandwidth of 0.66 GHz) to 15.1 GHz (with a bandwidth of 0.88 GHz). The simulated gain of the antenna in the “on” and “off” configuration is 3.0 and 3.7 dBi, respectively.

**Figure 10:**
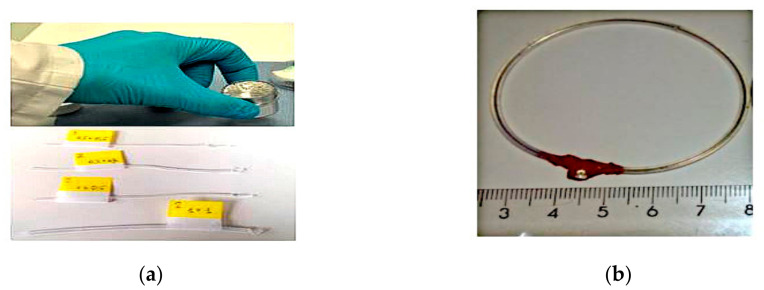
The proposed wearable antenna. (**a**) Galinstan and silicon tubing. (**b**) Loop antenna [47].

In another study presented in [47], a frequency-reconfigurable wearable antenna is proposed. This loop antenna was prototyped by injecting the Galinstan liquid metal into silicon tubing, as shown in Figure 10. Two conductive pins with silicon-based glue are inserted into the tubing ends to avoid the leakage of the liquid metal. This design operated at 868 MHz and can be tuned to 2.45 GHz with a stretching ability of around 150%. This approach features simplicity in prototyping, high flexibility, stretchability, and ease of integration into wearable devices.

#### 3.1.2. Reactive Loading Using Liquid Metals

**Figure 11:**
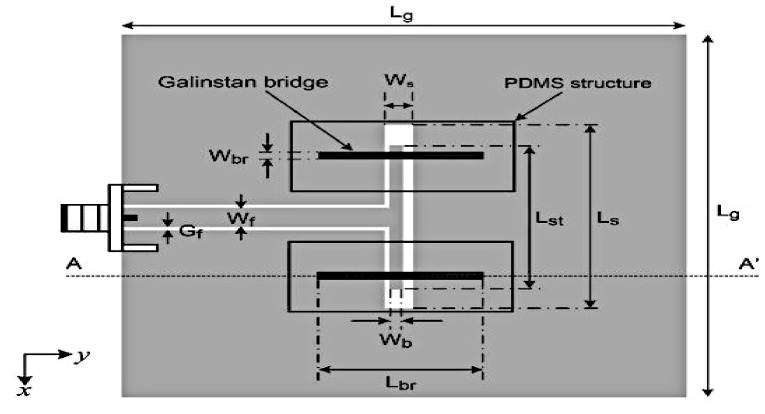
The CPW folded slot antenna with Galinstan bridges [23].

An example of the reactive loading technique involves the integration of Galinstan liquid metal into slot structures to achieve frequency reconfigurability in a coplanar waveguide (CPW)-fed folded slot antenna in [23]. In this design, two pairs of Galinstan-filled microchannels are used to achieve the frequency tuning of three operating frequencies, 2.4, 3.5, and 5.8 GHz with resonant gains of 1.2 dBi, as depicted in Figure 11. The two pairs of microfluidic channels are separated using two polydimethylsiloxane (PDMS) structures. Reactive loading is enabled by placing Galinstan bridges on top of the folded slot to tune the antenna frequency, offering size miniaturization, a very wide switching ranging from 2.4 to 5.8 GHz (switching ratio of more than 2.5:1). 

**Figure 12:**
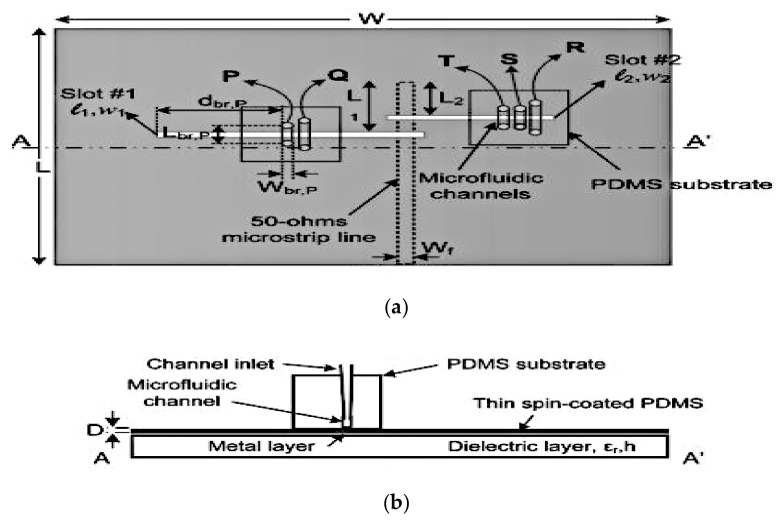
(**a**) Top view of the proposed switchable dual band slot antenna. (**b**) A-A’ cross section of the antenna [35].

On the other hand, the switchable dual band slot antenna proposed in [35] featured an overall frequency coverage ratio of 3:1 (1.8 to 5.4 GHz). In this design, five microchannels for Galinstan liquid metal are separated by two spins coated PDMS structures. By emptying/filling the Galinstan bridges illustrated in Figure 12, the antenna can be switched to operate in a dual band mode. The Galinstan bridges provide the reactive loading effect which can be used to independently control the operating frequency of the antenna. The proposed design provides a frequency tuning range of 1.8–3.1 GHz (in the first band) and 3.2–5.4 GHz (in the second band) with a high radiation efficiency of about 78% and 82%, respectively. The peak gain of antenna at its resonance ranges from 1.1 to 3.4 dBi.

**Figure 13:**
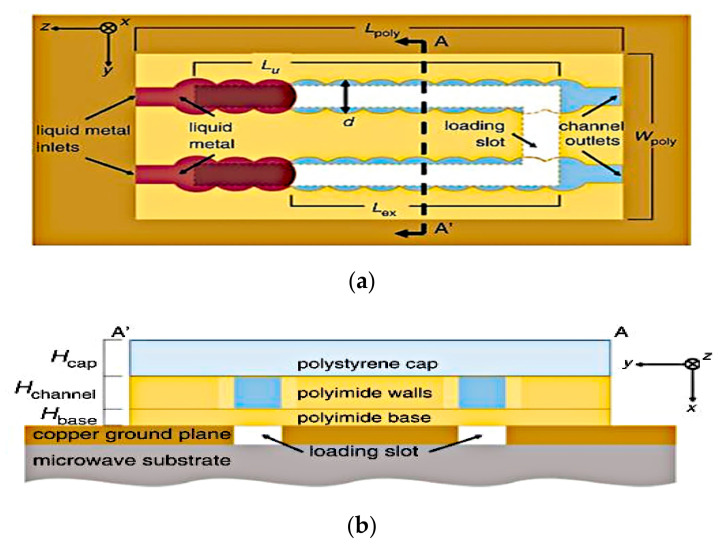
(**a**) Frequency-tunable antenna. (**b**) Cross section of the microfluidic channel structure [48].

In [48], a slotted patch antenna loaded using a pair of open-ended Galinstan channels is presented, as seen in Figure 13. The pair of open-ended channels is placed directly at the end of the U-shaped slot. The liquid channels are located near the ground plane to avoid them affecting the desired antenna radiation. Altering the length of the Galinstan filled channel changes the resonant frequency of the antenna. Besides that, the microfluidic channels are formed as interlocking circles to facilitate the control the resting position of Galinstan. Results indicate that the reactive loading from the U-slot and channels enabled bandwidth tuning of up to 11.2%, between 1.85 and 2.07 GHz with at least 2.1 dBi to a maximum of 4.1 dBi.

#### 3.1.3. Other Frequency Reconfiguration Techniques

**Figure 14:**
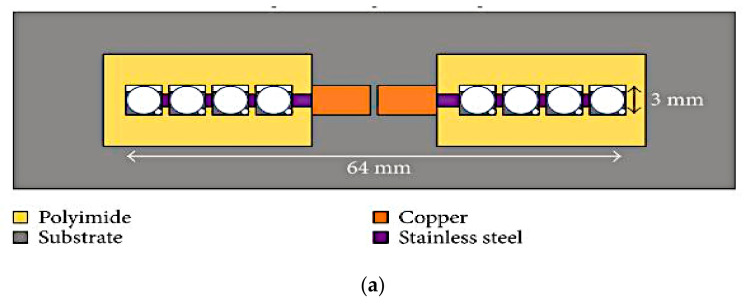

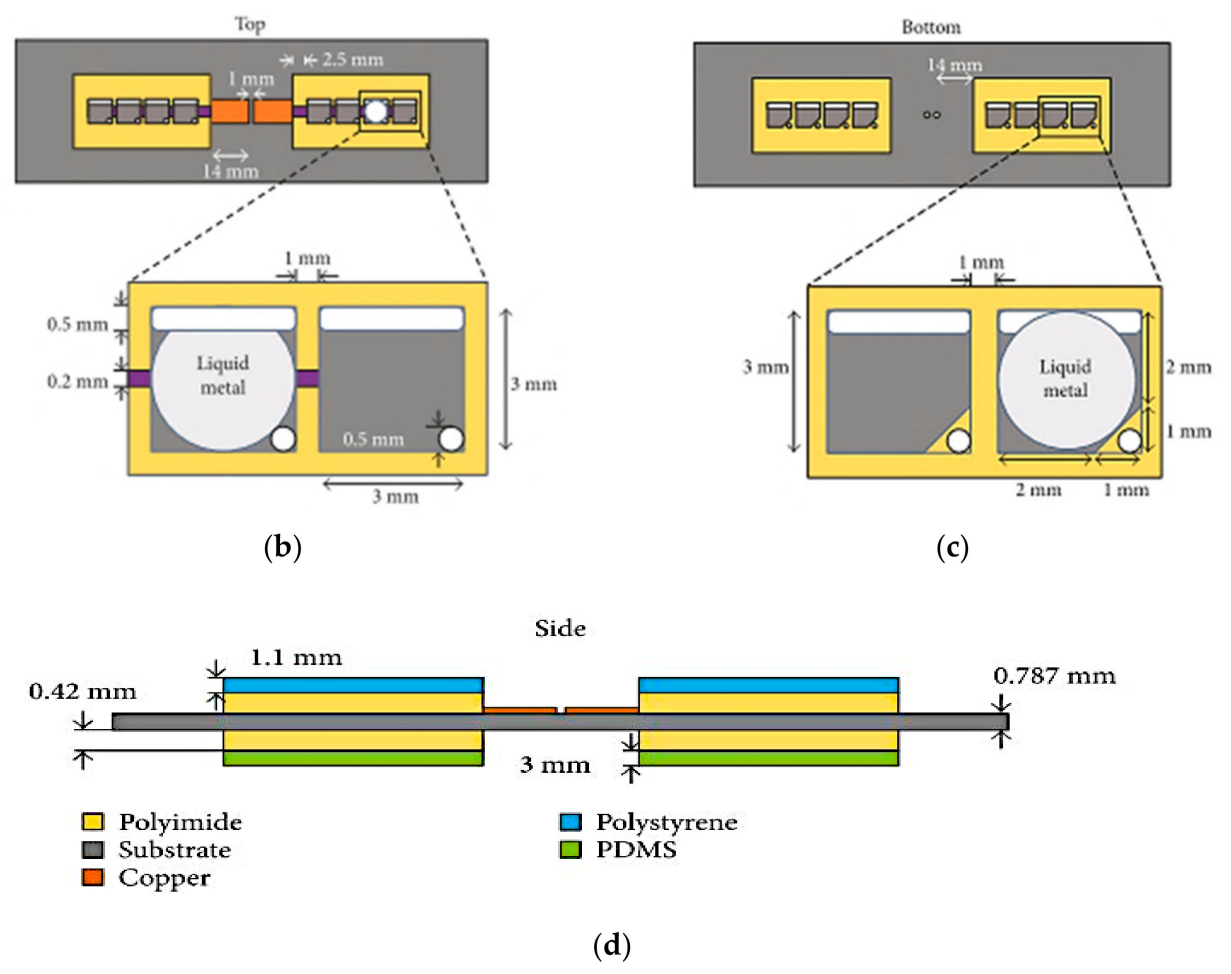
(**a**) The pixelated dipole antenna. (**b**) Top side pixel: ‘on’ state on the left side. (**c**) Bottom side pixel: ‘off’ state on the left side. (**d**) Side view [43].

Besides the two commonly used frequency reconfiguration techniques, there are several other methods which can be used for the same purpose. The first example is the pixelated dipole antenna illustrated in Figure 14 [43]. This antenna is designed based on a planar dipole with two side arms. Each arm is replaced by a 1 × 4 pixel array and is connected to the copper section through a soldered wire. Three additional materials are used to fabricate this antenna; polyimide was used to build the pixel walls, polystyrene as the top cover for the pixel, and PDMS as the bottom cover of the pixel array. To connect adjacent pixels, stainless-steel connectors are embedded between the pixel walls. For frequency reconfigurability, these pixels are turned on by electrically actuating the Galinstan from the reservoir below the antenna into the pixel. On the contrary, the pixel can be turned off by withdrawing the liquid metal back into the reservoir. This approach resulted in the antenna’s capability of switching to four different resonant frequencies: at 1.68, 1.85, 2.12, and 2.51 GHz, with radiation efficiency ranging from 70.2% to 75.4% and a peak gain variation of approximately ±3 dBi. Results have also indicated that this pixelated antenna did not affect the radiation pattern at the resonant frequencies compared to the measurement of the baseline planar dipole made using copper.

**Figure 15:**
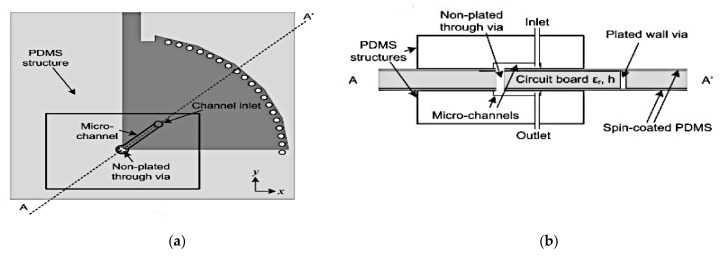

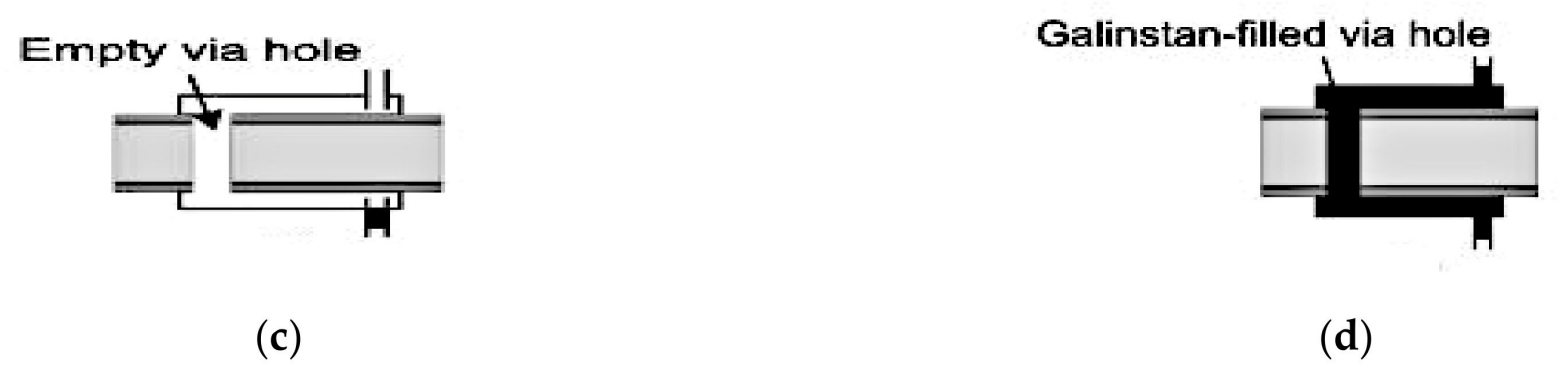
The proposed reconfigurable quarter-mode substrate integrated waveguide (QMSIW) antenna. (**a**) Top view. (**b**) A-A’ cross section view. (**c**,**d**) ON and OFF states of via [49].

Next, a frequency-switchable antenna using metallic fluid and via was presented in [49]. The switchable antenna is designed based on a quarter-mode substrate integrated waveguide (QMSIV). This antenna is capable of frequency tuning from 3.2 to 4.7 GHz with a frequency switching ratio of 1.45:1. To fabricate the antenna, the printed circuit board (PCB) technology, 3D printing and soft-lithography techniques were used to bond the PDMS structure to the circuit board of the QMSIW. This design employed a nonplated through via at the corners of the QMSIW, as shown in Figure 15. Both the top and bottom fluidic channels are connected using nonplated via holes, which can either be filled with Galinstan or emptied. When emptied, the antenna produced a resonant frequency of about 3.2 GHz, and this is increased when it is filled with Galinstan. The maximum measured gain for the disconnected and connected corner vias using Galinstan is 4.6 and 5 dBi, respectively.

**Figure 16:**
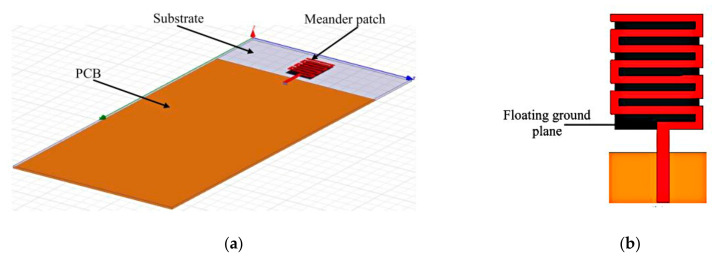
The meander antenna. (**a**) Perspective view. (**b**) Meander patch [50].

In [50], a reconfigurable meander antenna was designed to provide operation from 0.5 to 3 GHz. As observed in Figure 16, the meandered patch is fabricated on top of the substrate, whereas a floating ground plane cavity is located on its reverse side, under this patch. Galinstan is then injected into the cavity of the floating ground plane to modify the electromagnetic coupling to the radiating patch, thus increasing its electrical length. This enabled frequency to be reconfigured with a radiation efficiency of more than 60% and a maximum realized gain of 2.77, 3.12, and 1.32 dBi at the resonant frequency of 0.93, 1.78, and 2.85 GHz, respectively.

A summary of frequency reconfigurable antenna techniques using conductive liquids is presented in Table 2. Most of the antenna designs in [22,23,32,35,45,47,48,49,50] have demonstrated frequency reconfiguration using manual actuation using syringe, whereas electrical actuation using CEW are used in [42,43,46] to achieve reconfigurability. While it is obvious that the former requires longer time to complete compared to the latter, manual actuation is simpler to demonstrate [36]. The majority of these studies have also integrated liquid-fillable slots to reconfigure frequency by the size modifications, resulting in the increase or decrease in electrical lengths. For instance, in [36], the fluidic channel is placed on the slotline and feedline to offer a tuning bandwidth of 26%, producing a gain ranging from 4.1 to 4.8 dBi. Actuation of the liquid metal is realized using pressure-driven air bubbles. On the other hand, fluidic channels can be integrated as slotlines, as proposed in [42]. This antenna featured 36 % of tuning bandwidth, and more importantly, a fully sealed structure for antenna robustness. On the contrary, electrolytes can be used to reconfigure antennas with open-ended fluidic channels, as proposed in [48]. However, such structural design may potentially result in leakage of liquid metal when tipped on its side, affecting the antenna robustness [42].

Despite that, 11.2% of tuning bandwidth can still be offered by such antenna, with a gain ranging from 2.1 to 4.1 dBi. Frequency reconfigurable antennas using liquid metal has also been proposed to alleviate the effects of users’ hands in mobile terminals. An example is the PIFA with liquid metal designed for operation in the low frequency range (698–746 MHz) in [22]. On the other hand, antennas with a wide bandwidth (ranging from 2.2 to 9.3 GHz) can be designed using shaped liquid metal, as highlighted in [32]. On the other hand, a double switchable patch operated between 14.2 and 15.1 GHz in [46] resulted in a moderate gain from 3 to 3.7 dBi. When implemented on stretchable materials, the reconfigurable antenna in [47] achieved a 150% stretching elasticity. Increasing the number of fluidic channels and properly designing them into antennas such as in [23,35] improved gain from 1.2 dBi to a maximum of 3.4 dBi. From the table, the frequency reconfigurable antenna with the highest gain is from [49] with 5 dBi, whereas the antenna in [45] yielded the highest tuning ratio of 70% compared to other fluidic antennas designed for frequency reconfiguration. It is also demonstrated in [43] that the lowest operating frequency which can be achieved for such antennas is between 1.68 and 2.51 by lengthening each arm of the pixelated dipole. Finally, at least 60% of radiation efficiency can be achieved by these liquid-based antennas, as shown in [35,43,50].

### 3.2. Polarization Reconfigurable Antennas

In this section, examples of different types of polarization-reconfigurable antennas will be presented. The employed approaches include the use of dielectric resonator antennas (DRAs), incorporating apertures in antennas, patches with liquid cavities, liquid notches in antennas, liquid-based switches in patches, liquid-filled parallel slots, and slotted patches with metasurface.

**Figure 17:**
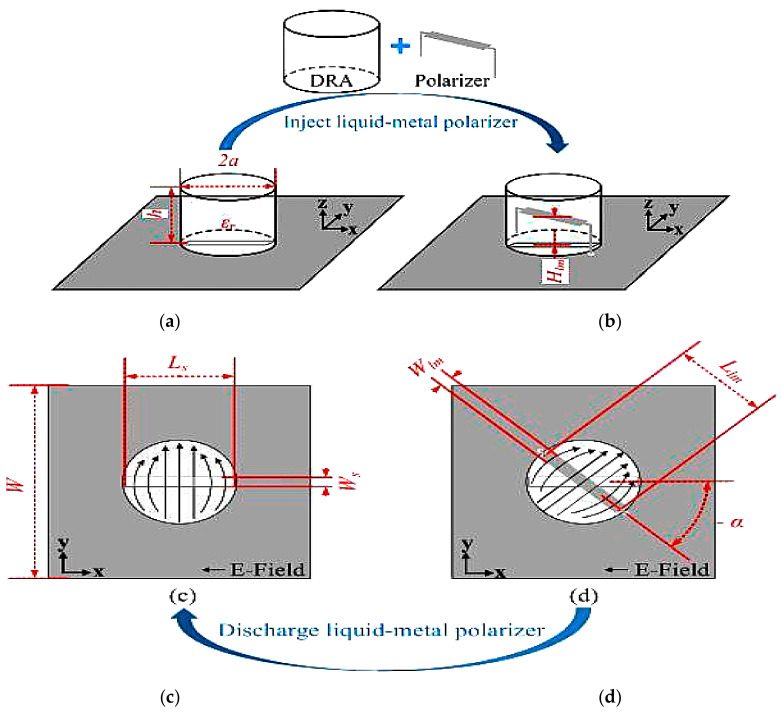

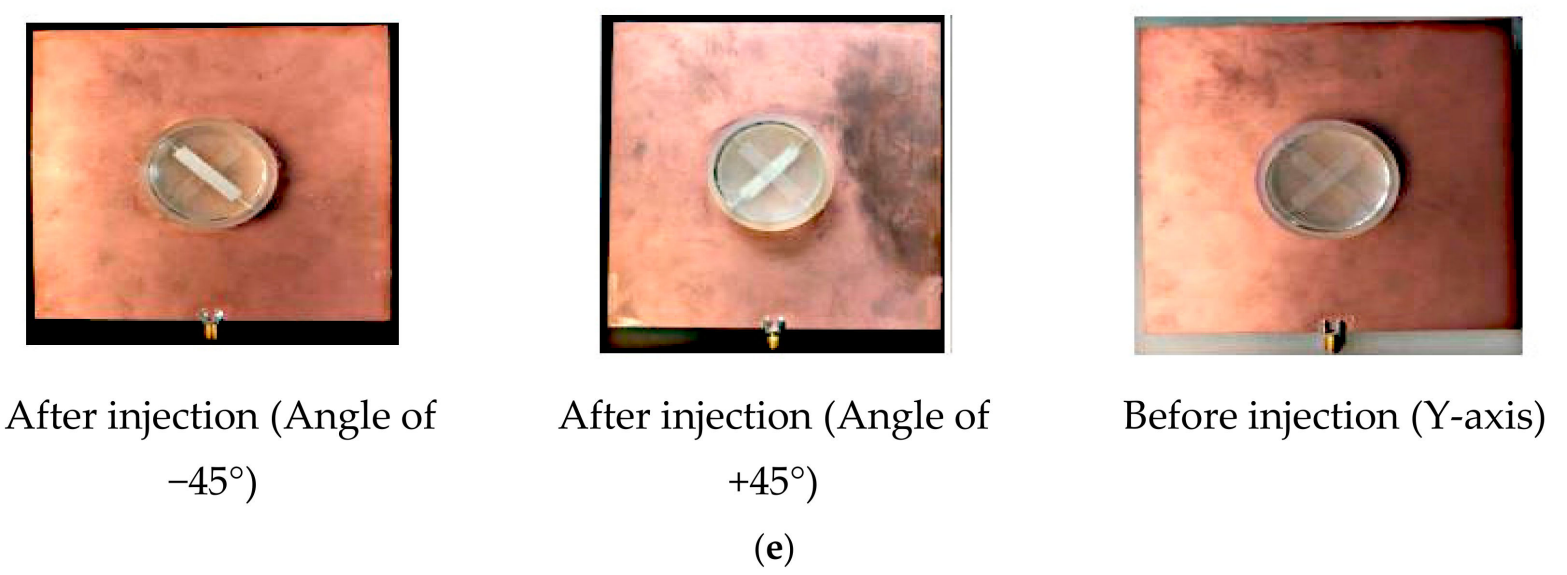
Design principles of the proposed DRA in [51]: (**a**) DRA without polarizer; (**b**) DRA with polarizer; (**c**) distribution of electric-field into DRA; (**d**) distribution of electric-field into DRA with polarizer; (**e**) working angle before and after the liquid-metal injection.

For the first example in [51], a polarization reconfigurable antenna using glass dielectric resonator filled with liquid metal is proposed as polarizer. This antenna illustrated in Figure 17 is designed to switch between three different polarizations at the *y*-axis: −45°, +45° and 0° at 2.4 GHz. A glass DRA integrated with an aperture is mounted above the ground plane. Electric field is fed from a microstrip transmission line through the aperture and into the DRA, polarizing the antenna along the *y*-axis. Next, liquid metal is injected into the glass DRA to distribute the electromagnetic field from the *y*-axis to the angle *α*. The liquid-metal polarizer rotates the angle of the electromagnetic field clockwise, as shown in Figure 17e. This design offers a wide bandwidth of 18% and a high radiation efficiency of more than 80%. In addition to this, the measured gain before and after injection are higher than 6 dBi, producing a broadside radiation pattern for both ±45° angles. 

**Figure 18:**
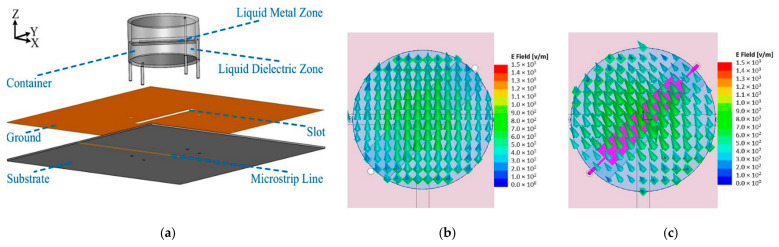
(**a**) Antenna structure. (**b**) Electric field of the dielectric resonator antenna (DRA) without the liquid metal. (**c**) Electric field of the dielectric resonator antenna (DRA) with the liquid metal [52].

Next, a liquid-filled DRA with radiation efficiency of more than 70% is designed for polarization reconfigurability, as shown in Figure 18 [52]. Ethyl acetate and Galinstan are used as the liquid dielectric solution and liquid-metal solution, respectively. The electric field of the main radiator (formed using the liquid DRA) is excited by an aperture, thus orientating it along the *y*-axis. Injection of the liquid metal into the liquid DRA will rotate the electric field from the *y*-axis towards 45° at 2.4 GHz, as shown in Figure 18b,c. A peak gain of 2–4 dBi and broadside radiation pattern for both states (angle of 90° and 45°) are demonstrated.

**Figure 19:**
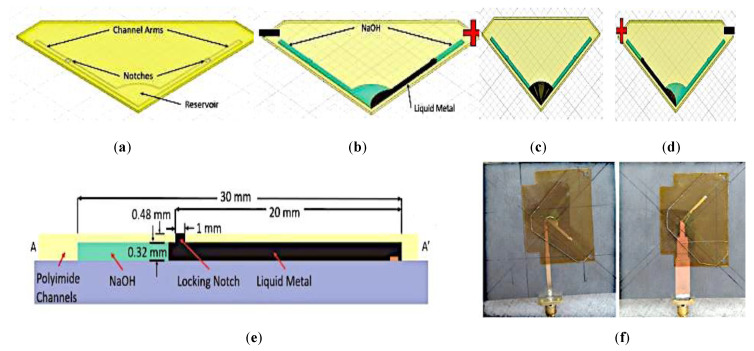
Liquid metal actuation within the fluidic channels for the antenna in [40]: (**a**) one-sided channel structure. (**b**) State 1 with liquid metal (black) and electrolyte solution (green) in the channels. (**c**) Withdrawing the liquid metal into reservoir. (**d**) Reversing the polarity to actuate the liquid metal into State 2. (**e**) The cross section of each arm structure. (**f**) Front and back of the assembled antenna.

Another antenna proposed in [40] provides polarization reconfigurability using an antipodal dipole antenna, as shown in Figure 19. Polarization can be switched using low-power electrical actuation of the liquid metal into states 1, 2, and 3. These states enable the antenna polarization to be oriented +45° from the feed, −45° from the feed, or be in the off state, respectively. Positive and negative voltage is applied to actuate the liquid metal into State 1. Conversely, this voltage difference will be minimal once the liquid metal has arrived at the notches in the channels. Reversing the applied bias voltage releases the metastable locking, causing the liquid metal to return into the reservoir. Metastable locking is used to maintain the operating state without the continuous application of voltage. Furthermore, the compact channels can reduce the effect of the electrolyte on the antenna radiation. The maximum gain achieved with the electrolyte solution is between 1.8 and 2.3 dBi, and between 2.4 and 3.0 dBi without the electrolyte. 

**Figure 20:**
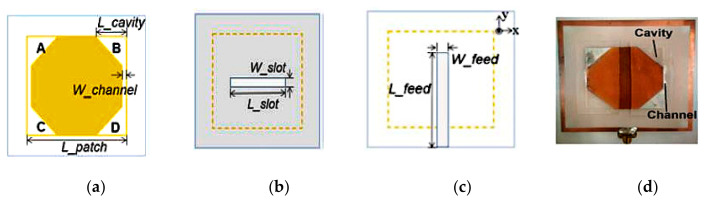
Schematic of antenna proposed in [53]: (**a**) upper layer, (**b**) top view, (**c**) bottom view of the lower layer, (**d**) top view of fabricated antenna.

Another approach presented in [53] offered a high radiation efficiency of more than 90%. This is done by employing elastomer, thus minimizing the liquid metal required. Different polarization states such as linear polarization (LP), left-hand circular polarization (LHCP), or right-hand circular polarization (RHCP) can be switched at 2.45 GHz by controlling the location of liquid metal in the four cavities. Standard printed circuit board is used to fabricate the bottom layer of the antenna, whereas the upper layer employed soft lithography technique to mold the cavities into the desired shapes. A truncated-corner square patch was selected to be fabricated using copper tapes and placed on the substrate, whereas Ecoflex is bonded with polyethylene terephthalate (PET) film and is placed conformal to the substrate to form the cavities (Figure 20). The measured impedance bandwidths (IBWs) are 23.2%, 33.6%, and 36.3% and the broadside gains are 7.24 dBi, 7.25 dBiC, and 7.33 dBiC for LP, LHCP, and RHCP, respectively. Moreover, the axial ratio bandwidths (ARBWs) for LHCP and RHCP are 3.06% (2.35–2.43 GHz) and 4.08% (2.38–2.48 GHz).

**Figure 21:**
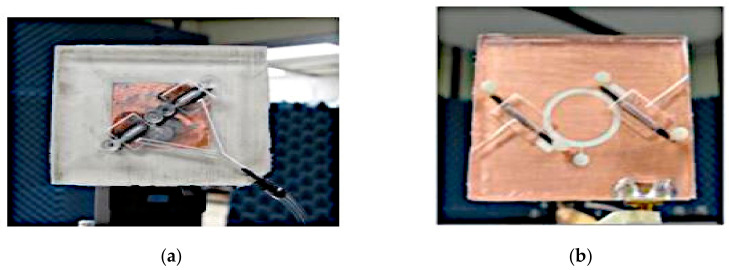
(**a**) Truncated-corner patch antenna. (**b**) Annular slot antenna [54].

On the other hand, a pressure-actuated switching mechanism is presented in [54] where EGaIn liquid metal was used to change the polarization of two antenna types. To demonstrate switchable capability between LP and CP, pneumatic actuation is applied onto (i) a truncated-corner patch antenna; (ii) an annular slot antenna, as shown in Figure 21. To contain the liquid, PDMS is bonded to a Rogers 4003 substrate, with two liquid metal channels and two air channels being integrated with the structure. In the first truncated-corner patch antenna, EGaIn is used to perform the switching between linear and circular polarizations. Pressure actuation of the two fluidic metal switches connected the patch and the parasitic elements located at the corners directly, resulting in a linear polarization. By releasing the pressure, the liquid metal is retracted into the middle section of the antenna, thus producing circular polarization. Meanwhile, in the second antenna designed based on an annular slot, the shorting across the slot line by the liquid generates a linear polarization. Conversely, retracting the liquid metal into the reservoir generates the circular polarization.

**Figure 22:**
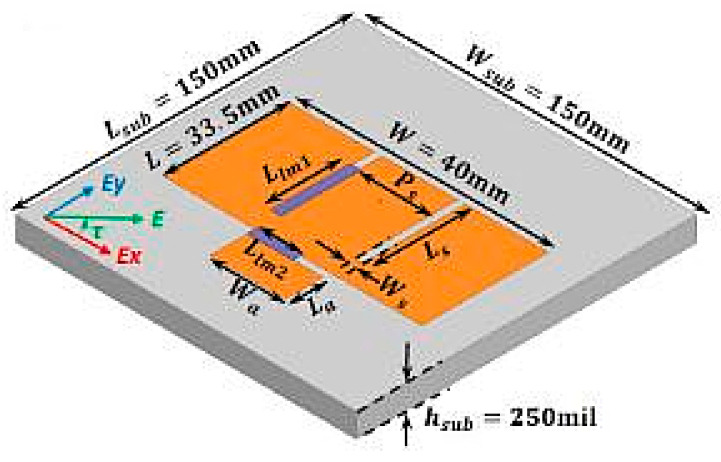
Extended E-shaped patch antenna [45].

Another example of polarization reconfigurable antenna is an E-shaped slotted patch antenna with an extended patch presented in [45] and illustrated in Figure 22. The microfluidic channels are integrated on the main patch and are allowed to flow between the slot of the main and extended patches to produce different polarizations; RHCP, LHCP, and LP. The length of the microfluidic channel *L*_lm1_ and *L*_lm2_ is tuned to control the orientation of the LP waves. The liquid metal can also be switched from *L*_lm1_ to the other parallel slots to obtain a 180° polarization state. Most importantly, a radiation efficiency above 98% was achieved via this design.

A summary of the polarization reconfigurable antennas using conductive liquid metals is presented in Table 3. It is found that the reconfigurable antennas with linear polarization designed using liquid are capable of switching from 0° to two orthogonal states, ±45° [51], 90° to 45° [52], and 45° to −45° [40]. These antennas have been designed based on the DRA and antipodal dipole topologies. On the other hand, a DRA incorporating liquid metal and dielectric liquid presented in [52] produced a 45° linear polarization. Its gain ranges from 2 to 4 dBi, with an antenna efficiency of more than 70%. Integrating this design with liquid metal polarizer [51] further improved the gain and efficiency to more than 6 dBi and 80%, respectively, compared to the antenna performance reported in [52]. On the other hand, the gain offered by the antipodal dipole in [40] is less compared to the DRA antennas in [51,52]. It can be seen that three types of polarizations including LP, LHCP, and RHCP can be realized, as demonstrated in [45,53]. The antenna in [45] offered the highest efficiency of more than 98% compared to other designs. Meanwhile, the aperture-coupled patch antenna in [53] produced a peak gain of 7.24 dBi, 7.25 dBiC, and 7.33 dBiC when operating in the LP, LHCP, and RHCP mode, respectively. Polarization switchability between LP and CP can also be realized using liquid-based reconfigurable antennas, as seen in [54]. However, its gain and efficiency are not reported. Finally, it must be noted that most of the antennas presented in the table implemented manual liquid actuation to achieve polarization reconfigurability, except for [40] which applied electrical actuation. 

### 3.3. Other Reconfigurations

This section presents the various other antennas which can be reconfigured in terms of gain, directivity, phase, and the combination of them (compound reconfiguration). Gain reconfiguration can be achieved by either changing or shifting the position of liquid stubs integrated into a patch antenna or by varying the number of liquid turns of a helical antenna. On the other hand, manipulating the height of the liquid elastomer in a two-arm spiral antenna enables directivity reconfiguration, whereas the rotation of the radiating element can produce phase reconfiguration. Compound reconfiguration can be achieved in helical structures and dipole antennas (polarization and pattern reconfiguration), changing the lengths of the arms of a crossed dipole antenna (frequency and polarization reconfiguration) and switching between helical and zigzag antenna (frequency, polarization, and pattern reconfiguration). More details of their concepts will be presented in the following subsections.

#### 3.3.1. Gain and Directivity Reconfigurable Antennas

**Figure 23:**
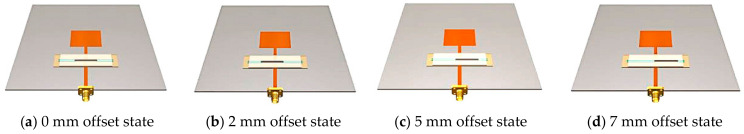
States of the Galinstan slug. (**a**) The 0 mm offset state. (**b**–**d**) The *X*-mm-offset state [17].

An example of gain reconfigurability is by electrically actuating liquid metal placed in a slug using the CEW technique, as proposed in [17]. This slug is positioned at *X* mm from the right of the microstrip feed line, which is also the midpoint of the liquid metal slug, as shown in Figure 23. The antenna is reconfigured by shifting the position of the Galinstan slug, thus tuning its gain from −5.90 to 4.43 dBi at 5 GHz, translating to a 10.33 dB analogue gain tuning. However, such simple tuning mechanism is unable to hold the liquid metal in place against unintentional movements.

**Figure 24:**
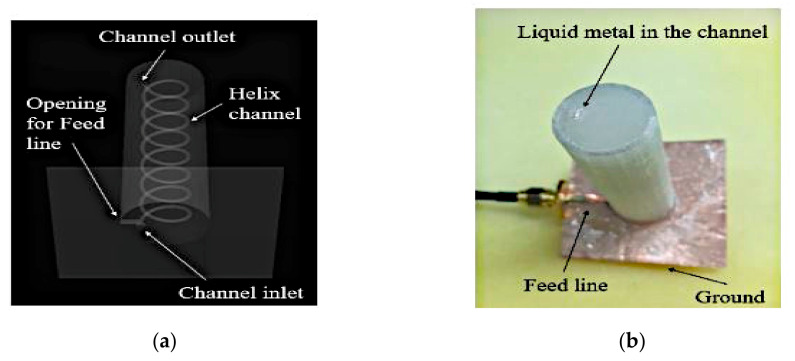
Fabricated prototype. (**a**) Photograph of antenna. (**b**) Proposed antenna [55].

Next, a reconfigurable helical antenna is presented in Figure 24 [55]. A cylinder containing helical channels is placed on top of a square copper patch. Tuning is achieved by gradually filling the 3D printed helical channels with EGaIn. Since the antenna gain is determined by the number helical turns, gain is effectively controlled by the volume of EGaIn that flowed into the channels. Increasing the number of helical turns from 2 to 8 increases the gain from about 5 to 9 dBi at 5 GHz.

**Figure 25:**
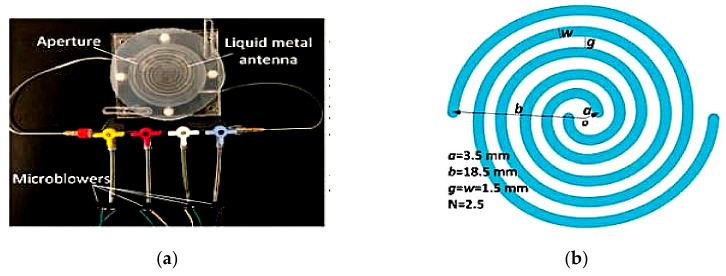
(**a**) The directivity-reconfigurable wideband antenna assembly. (**b**) Design parameters of the two-arm spiral antenna [16].

On the other hand, directivity reconfiguration can be achieved using a two-arm spiral antenna [16]. EGaIn liquid metal is enclosed into a stretchable silicone elastomer to form a wideband antenna, as seen in Figure 25. By inflating using a microblower, the height of the elastomer in the *y*-direction (90°) will be increased, simultaneously increasing its main lobe directivity (in the *y*-direction) and decreasing the radiation along -*y*-direction (270°). This antenna generates a circular polarization and enables a wideband operation from 6.9 to 13.8 GHz, with a radiation efficiency between 40% and 72%.

#### 3.3.2. Phase Reconfigurable Antenna

**Figure 26:**
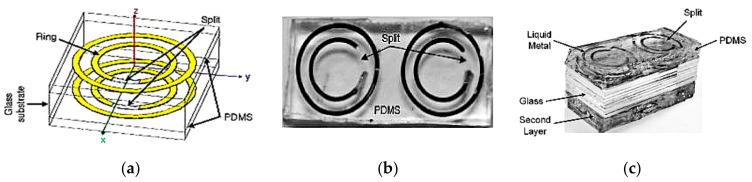
Unit cell of the nested ring-split ring transmitarray. (**a**) Double layered. (**b**) One layer for the rotation angle of 20°. (**c**) Fabricated double layered antenna [56].

Besides gain, the phase of an antenna can also be reconfigured using liquid metals. One of the first studies presenting such reconfigurability is a microfluidic transmitarray unit cell presented in [56]. Continuous phase shifting is achieved using the element rotation method. The unit cell consists of a double-layered nested ring-split ring structure implemented in the form of microfluidic channels, and are integrated within a PDMS substrate, as shown in Figure 26. Galinstan is injected into the rings to form the conductive regions, whereas the split regions are air-filled. Moving the liquid metal around the ring along the split offers a 360° range of linear phase shift through the unit cell in the transmitted field. Its main advantage is that each unit cell can be easily controlled by a pair of tubes attached to a micropump.

#### 3.3.3. Compound Reconfigurable Antennas

There are various enabling technologies to combine two or more parameters reconfiguration in an antenna. They may differ significantly in terms of efficiency, performance, or other characteristics such as integration and compactness.

**Figure 27:**
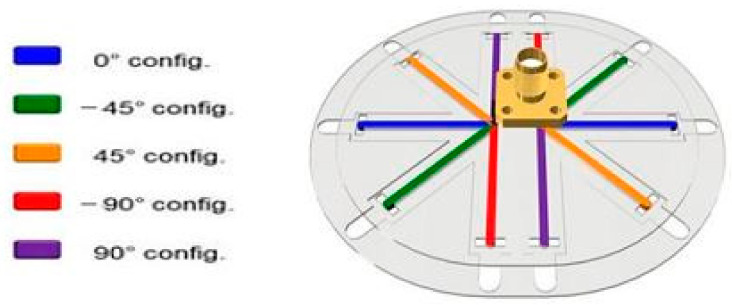
Polarization and pattern reconfigurable dipole antenna [41].

The first example of such compound reconfigurable antenna is presented in [41], where the antenna is capable of polarization and pattern reconfigurability using ECA. Five discrete phase shift states can be provided, i.e., at 0°, −45°, 45°, −90°, and 90° to enable polarization reconfigurability and null directions. To obtain X° configuration, the liquid metal is electrically actuated into the (colored) fluid arms which are enclosed by polyimide fixture (grey), as shown in Figure 27. This antenna achieved a radiation efficiency ranging from 69% to 97%. On the other hand, the local polyimide-built surface energy is used to enable metastable locking, which is needed to maintain the fluid configuration in each state.

**Figure 28:**
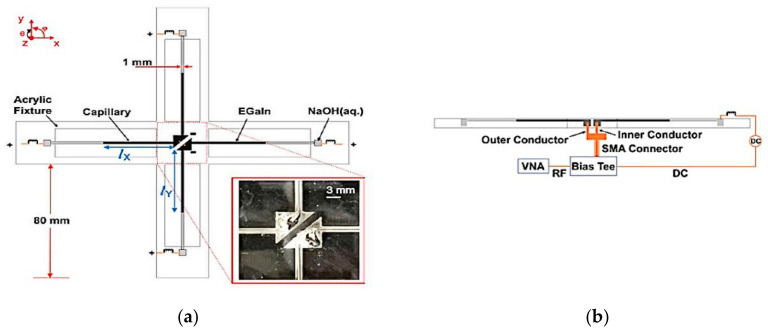
Schematic of the (**a**) reconfigurable crossed dipole, and (**b**) feed detail [18].

Next, a crossed dipole with reconfigurable frequency and polarization is proposed in [18]. It comprises of four glass capillaries designed in an acrylic fixture, as shown in Figure 28. Six laser-cut cavities in the acrylic fixture form two central liquid metal reservoirs and four outer electrolyte reservoirs to support both ends of the capillaries. DC voltage is used to shorten and lengthen the liquid metal in the capillaries to implement the ECC technique. The multidirectional spread of the liquid metal changes the length of the dipole arms to generate the linear and switchable linear (from 0.8 to 3 GHz) to circular polarization (from 0.59 to 1.63 GHz), with a radiation efficiency ranging from 41% to 70%, similar to [37].

**Figure 29:**
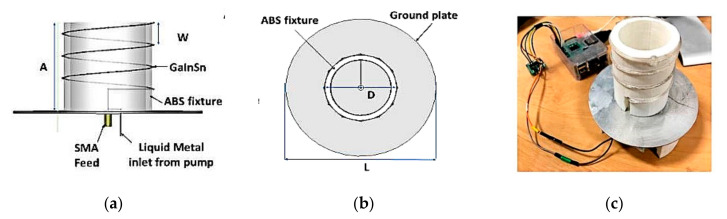
The proposed antenna in [39]: (**a**) side view, (**b**) top view, and (**c**) antenna prototype.

Besides that, a pattern and polarization-reconfigurable helical antenna using mechanically actuated liquid metal is shown in Figure 29 [39]. The antenna comprises a helical structure made using a polymer tube, which is wound around an Acrylonitrile Butadiene Styrene (ABS) fixture. Galinstan liquid metal is pumped into the polymer tube using a Raspberry Pi-controlled micropump. This 1.575 GHz antenna generated four beams: circularly and elliptically polarized axial beams, and linearly polarized semidoughnut and axial beams with their peak gains of 8.5, 7.6, 1.1, and 5.9 dBi, respectively.

**Figure 30:**
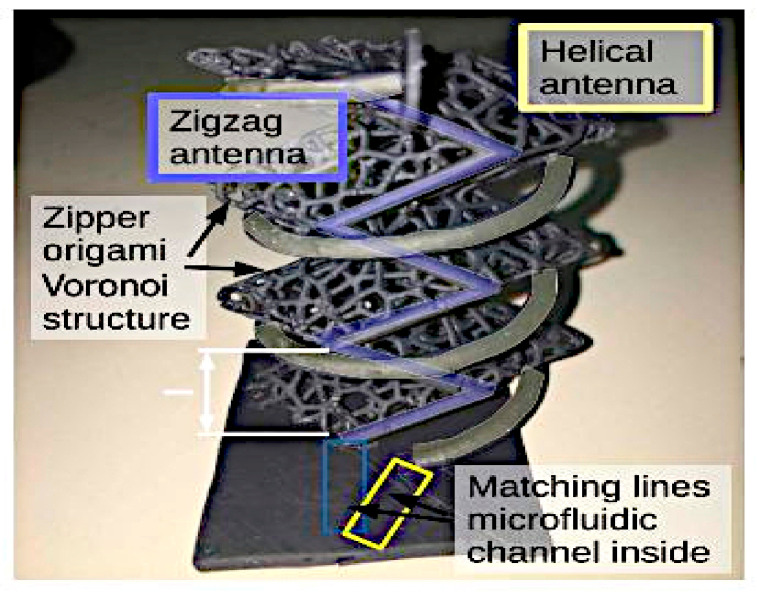
3D printed antenna tree [57].

In [57], a 3D printed ‘tree’ antenna was designed for frequency, polarization, and radiation pattern reconfiguration. A zig-zag antenna and a helical antenna are integrated into a zipper origami Voronoi structure, as shown in Figure 30. This structure is used as the scaffolding to mechanically tune the radiation pattern and to minimize the storage requirement. EGaIn is used to switch between the two antennas and to allow the implementation of tunability. The compression of this ‘tree’ (zig-zag and helical) antenna produces a dual band operation in the 3G and 5G bands, with dual polarization and with directional and omnidirectional radiation patterns. 

Antennas implementing conductive liquid for other types of reconfiguration are summarized in Table 4. Gain reconfigurability can generally be achieved by implementing liquid metal slug in patch antennas by correctly choosing its tuning position. In the case of [17], this coincides with the center microstrip feed line. This resulted in a large gain tuning of 10.33 dB, from −5.9 to 4.43 dB. On the other hand, gain tuning of more than 4 dBi can be implemented by changing the number of helical turns, as demonstrated in [55]. Furthermore, antenna directivity can also be switched using liquid metals for the case of a spiral antenna in [16]. The two arms of this spiral antenna are gradually filled with liquid to enable directivity switch from 90° to 270° with an efficiency of up to 72%. Finally, the phase shift caused by unit cells can be linearly reconfigured by implementing liquid metal-filled split ring elements, which was demonstrated for a transmitarray in [56]. Besides single parameter reconfiguration, compound reconfiguration offers a greater flexibility for future communication systems. Among the types of reconfiguration demonstrated using liquid metals include the filling of dipole arms [41] and LM helical turns [39] to produce the polarization and pattern reconfiguration. Beams can be focused towards either the 0°, ±45°, or ±90° directions, as seen in [41], whereas four types of different polarizations (circular-, elliptical axial, linear axial, and linear) with gains of up to 8.5 dBi have been reported [39]. Meanwhile, frequency and polarization reconfiguration can be generated by the liquid filled antenna in [18], with a tuning capability from 0.8 to 3 GHz (operating in LP) and from 0.89 to 1.63 GHz (operating in CP). Its efficiency produced is more than 40 %. Finally, frequency, direction, and polarization reconfiguration are enabled via the combination of two different antennas (zig-zag and helical) into a single structure [57]. This antenna has generated LP at 3 GHz with a directional radiation pattern, and CP at 5 GHz with an omnidirectional radiation pattern. 

## 4. Reconfigurable Antennas Using Other Liquids

**Figure 31:**
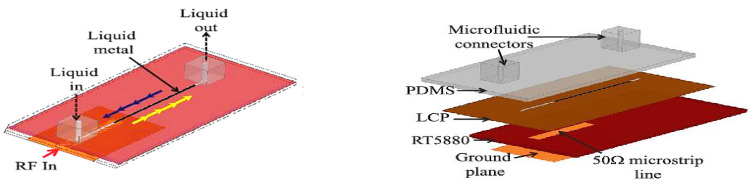
Substrate stack-up of the liquid-metal monopole antenna [38].

In this section, reconfigurable technologies using other liquids which enables frequency, polarization, and pattern reconfiguration will be presented. Similarly, the main principles remain the same—changes to the antenna physical dimensions enables frequency reconfiguration. On the other hand, the use of DRAs enabled polarization and pattern reconfigurations. The first example is a microfluidically reconfigurable frequency-tunable monopole antenna employing mercury as its conducting liquid [38]. Mercury is chosen for its low rate stiction and oxidation properties. A PDMS substrate containing microfluidic channels is designed to form a monopole antenna, as shown in Figure 31. To fabricate these channels, the lithography process is employed, and the completed PDMS layer is then sealed with a liquid crystal polymer (LCP) layer. This channel is aligned with the microstrip feedline to generate capacitive coupling through the LCP layer. This LCP layer is then bonded to a Rogers RT5880 substrate consisting of a microstrip line and a ground plane. Initially, a syringe was used to inject the mercury and Teflon solutions inside the microfluidic channel. This solution is moved within the substrate, on the microstrip feed line using a bidirectional micropump, which then reconfigures the physical length of the antenna. This antenna provided a frequency tuning range from 1.29 to 5.17 GHz with measured realized gain from 1.3 to 3 dB and radiation efficiency above 80%. The proposed antenna can also be used to form a monopole array antenna capable of frequency tuning from 2.5 to 5 GHz with broadside gain of more than 6 dB and a radiation efficiency of 80% and 65%, respectively. The tuning speed ratio for monopole antenna to monopole array is about 2: 1 for 242.5 and 125 MHz/s, respectively.

**Figure 32:**
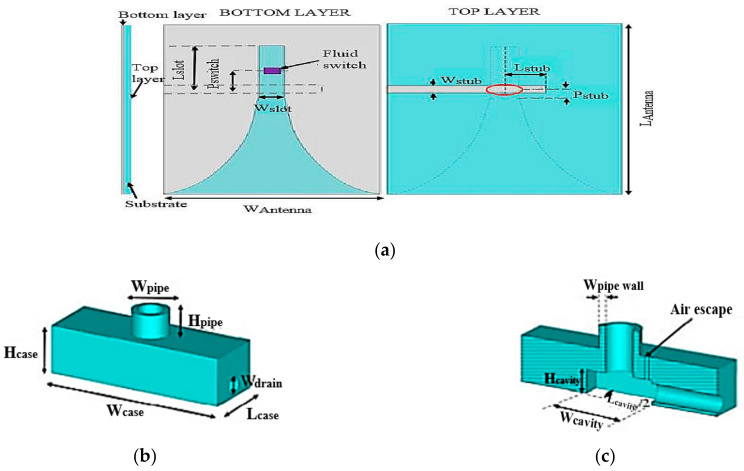
(**a**) Bottom and top layer of the fluidically switched antenna. (**b**) Fluid switch. (**c**) Cross sectional view of the fluid switch [58].

Next, a fluidically switched Vivaldi antenna using ionized water was proposed in [58], whose operation can be tuned between the 3.2 and 4.5 GHz band. A microstrip feed line with an open circuit stub is fabricated on the top layer of the substrate, as illustrated in Figure 32. The feed line couples the signal to the slot line on the bottom layer. The conductive fluid switch enclosure contains deionized water dissolved using 2 mol of potassium chloride (KCI) solution. Operation in the higher frequency band of 4.5 GHz is achieved by pumping conductive fluid into the switch to shorten the current path. On the other hand, draining out the fluid enables operation at 3.2 GHz with an efficiency of 87%. The measured gain for this antenna is 11 and 10.9 dBi at 3.2 and 4.5 GHz, respectively.

**Figure 33:**
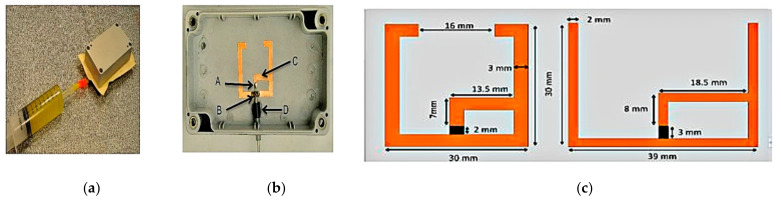
(**a**) Plastic antenna box, latex diaphragm, and syringe. (**b**) Bent dipole antenna and coaxial cable arrangement. (**c**) Dimensions of the two bent dipole antennas with castor oil (on the left) and ethyl acetate (on the right) [59].

In [59], a frequency-reconfigurable planar antenna using castor oil and ethyl acetate as its dielectric fluid has been proposed, as illustrated in Figure 33. A sealed plastic enclosure box made using ABS (as its body) and clear polycarbonate (as its lid) is used to electrically isolate the control circuitry from the copper sheet antenna. The bent dipole antenna is placed on the inner sides of the ABS enclosure. A flexible diaphragm comprising a latex rubber sheet is placed across the opening of the sealed box. To feed the antenna, a coaxial cable is inserted into the cavity, with the inner conductor soldered to point A, whereas the coaxial shielding is connected to point B. A gamma matching section made using ferrite beads (denoted as sections C and D) is designed to act as a choke to stop the unbalanced current from flowing down the cable. Pumping of the cavity on the polycarbonate side (behind the antenna) will bind the dielectric fluid onto the upper boundary of the flexible diaphragm, thus enabling frequency reconfiguration. By using the two dielectric fluids (castor oil and ethyl acetate), a tuning range of 25% (from 1.17 to 1.5 GHz) and 46% (from 0.9 to 1.44 GHz) can be achieved. This comes at a cost of a decrease in efficiency to 48% and 25%, respectively, in each band.

**Figure 34:**
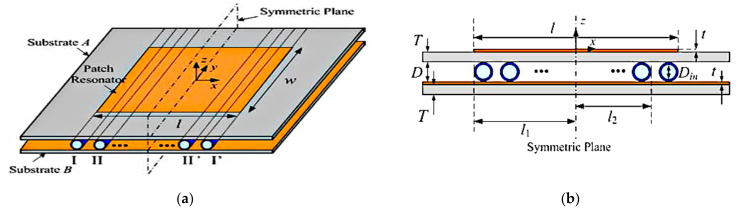
Microfluidic frequency reconfigurable patch antenna. (**a**) 3D view. (**b**) Side view [60].

Another example of a frequency reconfigurable patch antenna uses deionized (DI) water placed in microfluidic channels [60]. Figure 34 illustrates the topology of the antenna, consisting of two Rogers 4003C substrates and polypropylene tubes acting as the microfluidic channels. The patch is designed on top of substrate A, while the copper on top of substrate B acts as ground plane. The tubes are symmetrically placed between substrate A and the ground. A micropump is used to pump the DI water in or out of the tubes. The tuning range, states, and frequencies are realized by adjusting the number and location of the tubes. It is operated based on the changes in effective dielectric constant value between the patch and ground plane. The operating frequency without the tubes is 2.04 GHz, whereas insertion of the two pairs of tubes achieved a tuning frequency from 1.391 to 1.861 GHz with a measured gain of above 6.7 dBi and a radiation efficiency more than 68.8%.

**Figure 35:**
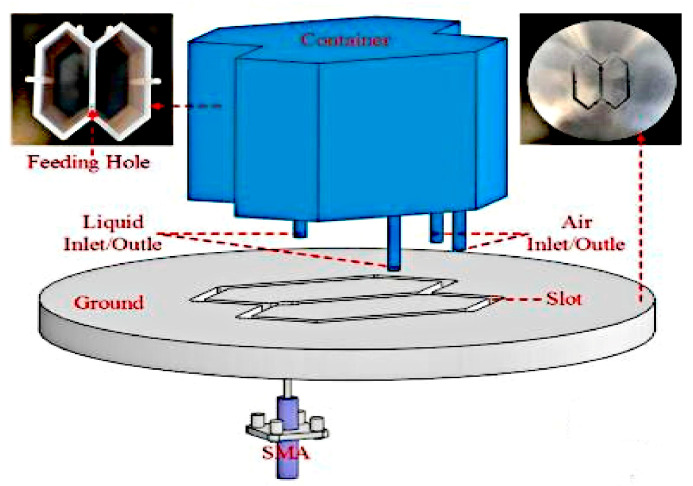
Liquid dielectric resonator antenna (DRA) [61].

In [61], an antenna with circular polarization reconfigurability is proposed using a liquid DRA. This antenna is designed for operation in the 2.4 GHz band for RFID applications. It is fed using a single probe and is integrated onto two arbitrary sides (left and right zones) of a 3D-printed container, as depicted in Figure 35. The polarization switching states can be changed by pumping the dielectric fluid (ethyl acetate) solution into the left or right section, resulting in a LHCP or RHCP, respectively. The proposed antenna featured an efficiency of more than 70%, a measured gain of higher than 2 dBiC, a wide operating bandwidth of 35.6% (from 2.08 to 2.98 GHz), and an axial ratio (AR) bandwidth of 16.3% (from 2.31 to 2.72 GHz).

**Figure 36:**
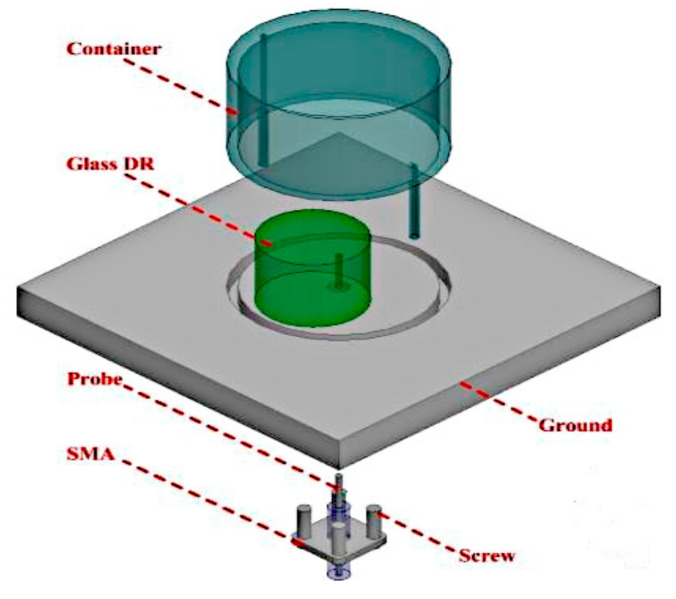
Reconfigurable dielectric resonator antenna (DRA) presented in [62].

Next, the application of dielectric fluid in tunable DRAs to achieve pattern reconfigurability is presented in [62]. This antenna made using two DRAs is illustrated in Figure 36. The glass cylinder represents the static inner zone of the DRA, whereas the outer zone is a liquid-based cylindrical DRA. A 3D-printed container is used to cover both DRAs, and both structures are fed using a coaxial probe. When dielectric fluid (ethyl acetate) is pumped in and out of the outer zone, the reconstituted DRA is excited in the conical transverse magnetic (TM_01*δ*_ mode), resulting in up to 60% of radiation efficiency. On the other hand, the glass DRA is excited in a broadside hybrid electromagnetic HEM_11*δ*_ mode, producing an efficiency more than 80%. Manipulating both DRA modes enables radiation pattern reconfigurability over a wide 35% impedance bandwidth, operating from 3.75 to 5.37 GHz with at least 3 dBi of measured gain. 

A summary of reconfigurable antennas using other liquids is presented in Table 5. Liquids used for reconfigurable antennas include mercury, deionized and ionized water, ethyl acetate, and castor oil. These liquids have been inserted into planar monopoles, dipoles, patch antennas, Vivaldi and DRAs, among others, to enable frequency, polarization, and radiation pattern reconfiguration. The frequency tuning realized using these liquids ranges from 0.9 to 1.44 GHz and from 1.17 to 1.5 GHz [59], from 1.391–1.861 GHz [60], from 3.2 and 4.5 GHz [58], and from 2.5 to 5 GHz [38]. The radiation efficiency and gain produced by these frequency-reconfigurable antennas are also topology- and material dependent. 

The monopole antenna in [38] produced a gain between 1.3 and 3 dB with an efficiency of 65–80%, whereas a gain of more than 6 dB is achieved when this monopole is modified into an array. Similarly, the Vivaldi antenna with fluidic switched using ionized water [58] produced an efficiency of 87%, but with a higher gain of about 10.9 dBi. The gain for a patch antenna when DI water-filled tubes are inserted between the ground and the patch is more than 6.7 dBi [60], with at least 68% of efficiency. Meanwhile, DRA-based reconfigurable antennas featured lower minimum radiation efficiencies, ranging between 60% [62] and 70% [61]. This property is intrinsic to DRAs due to the radiation from mostly dielectric materials in comparison to conventional antennas. It is important to note that these DRAs are not frequency reconfigurable, and instead are polarization (LHCP- to RHCP and vice versa) [61] or radiation pattern reconfigurable antennas [62]. Their gains are also limited to 2 dBiC and 3 dBi, respectively.

## 5. Future Perspectives

There are several practical considerations in maintaining and improving the performance of reconfigurable antennas using liquid metal. Some of the considerations are suggested in this section, based on the findings of the literature. The most prominent limitation of the liquid metal is the possibility of the oxidation layer build-up caused by the use of gallium-based liquid metal such as Galinstan and EGaIn. An effective solution in alleviating this is by using electrolytes. In addition to this, there are still challenges with the commonly used actuation methods such as ECA, CEW, and ECC. They include the need for suitable supporting electrolytes. Therefore, studies related to the viscosity levels of acids, alkaline, and bases must be performed to ensure the continual prevention of the oxidation layer. Liquid metal is also susceptible to the corrosive effects of copper probes. Thus, the actuating probe must be chosen with care to ensure its resistance to alkaline or acid corrosion. Finally, studies also must be conducted to evaluate the life expectancy of metal fluids and electrolytes. This is to ensure structure durability and stable antenna performance, which then translates to the overall cost effectiveness of such liquid-based reconfigurable antennas.

## 6. Conclusions

This manuscript reviewed the current state-of-the-art in liquid-based reconfigurable antennas. Reconfiguration of antenna parameters (such as frequency, polarization, gain, directivity, phase, or their combinations) can be achieved by controlling the flow/insertion of liquid metal into microfluidic channels to alter the physical size of antennas. The implementation of capacitive or reactive loading also can reduce the physical size of antenna, at the cost of antenna efficiency. Besides that, the use of lossy material also affects antenna efficiency. Straight fluidic channels have been mainly used to simultaneously ensure robustness of the antenna and to enable gradual tuning, which improves the positioning accuracy of the liquid. Nonetheless, channels with notches or interlocking circles have also been introduced to maintain the liquid metal in position against any antenna tilting or unintentional motions. On the contrary, open ended fluidic channels must be avoided as they do not securely maintain the liquid metal in the channel, are prone to leakage, and will affect antenna robustness.

From these recent studies, planar antennas are the most widely used antenna as frequency reconfigurable antennas. Such antenna types include the slotted antennas, PIFAs, planar patches, pixelated dipoles, switchable QMSIV, and meandered patch antennas. In addition to that, loop antennas which use fluidic tubes are another viable alternative. On the other hand, polarization reconfiguration is achieved using dipole antennas, truncated-corner patches, slotted antennas, and annular antennas. Besides that, the concepts of DRAs in enabling polarization reconfiguration is also presented and discussed. Meanwhile, gain and directivity reconfiguration can be achieved by tuning the length of the matching stub. To demonstrate the former concept, a two-arm spiral antenna is designed on a stretchable silicone elastomer to obtain directivity reconfigurability, whereas tunable unit cells in the latter shifts the phase for a transmitarray. The final category, which is the compound reconfigurable antenna can be implemented in practice by using dipole, crossed dipole, helical, or zig-zag antennas. These antennas are capable of reconfiguring two or more antenna parameters (frequency, polarization, gain, phase, etc.) instead of a single parameter. 

It can be summarized that there exist three methods can be used to control liquid flow and consequently reconfigure the antenna: (i) manual actuation, (ii) pneumatic actuation, and (iii) electrical actuation. Generally, a syringe is used to manually actuate liquid metal using air pressure. This process can be repeated and reversed by designating the inlet and outlet of the liquid metal separately. However, when using a syringe, additional time is taken for moving the fluid in the channels. Hence, pneumatic actuation via micropump can be used to significantly reduce this duration of actuation. However, in applications which require antennas to be compact in size, the use of micropump units is a disadvantage due to the additional space requirement. Furthermore, it can be challenging to practically implement frequency reconfigurable antenna arrays with high gains using such solution. 

For this reason, pump-free electrical potential actuation mechanisms are needed. These mechanisms include the likes of ECA, CEW, and ECC, which require the direct contact of electrolytes with the liquid metals. These techniques are low power, and they operate based on the manipulation of the surface tension of the electrolytes. The ECA actuation of liquid metals can be performed by applying a positive DC bias voltage on the electrolyte that surrounds the liquid metal to induce its surface tension. This then moves the liquid metal towards the positive bias. Conversely, swapping the polarities of the applied voltage will restore the liquid metal to its original position. To avoid the liquid metal from withdrawing back to its original position, a continuous bias voltage must be applied. As an alternative, ECA can be incorporated with the metastable locking method to sustain the position of the liquid metal without any continuous bias voltage. In contrast to ECA, the CEW method allows the liquid metal to move from point to point instead of deforming or reshaping. In ECA and CEW approaches, there is no chemical reaction involved in the actuation of liquid metal. However, neutral electrolytes can build up oxide layers and mechanically hinder the movements of liquid metal in these channels. Using the ECC method, excess oxidation layers can be removed using a strong acid or base solutions. 

Despite the significant progress in each approach, there are still ample possibilities for future studies and developments. As trade-off typically exists between the material properties of the liquids, types of channels, and antenna performance, novel antenna designs, new materials, innovative techniques in liquid metal actuation, and antenna fabrication, or any combination of them will continue to attract antenna researchers’ attention for years to come.

## Figures and Tables

**Table 1:** Summary of the properties of Galinstan and EGaIn, adapted from [25,34].

Properties	Galinstan	EGaIn
Material composition	Gallium (68.5%), indium (21.5%) and tin or stannum (10%).	Gallium (75.5%) and indium (24.5%)
Appearance at room temperature	Liquid	Liquid
Color	Silver	Silver
Melting point(°C)	−19	15.5
Boiling Point (°C)	>1300	2000
Viscosity (Pa.s)	2.4 × 10^−3^	2.0 × 10^−3^
Density (kg/m^3^)	6440	6280
Surface tension (N/m)	0.718	0.624
Electrical conductivity (S/m)	3.46 × 10^6^	3.4 × 10^6^
Thermal conductivity (W/m.K)	16.5	26.4

**Table 2:** Summary of frequency reconfigurable antennas using nontoxic conductive liquids.

Ref	Antenna Type	Approach	Liquid Metal/Actuation	Freq. Range (GHz)	Tuning BW (%)/Tuning Ratio	Gain (dB/dBi)/ Efficiency (%)
[36]	Slot	Size modification—slotline and feed line	Galinstan/manual-air bubble	1.42–1.84	26%	4.1–4.8 dBi
[42]	Slot	Size modification—slotline	Galinstan/electrical- continuous electrowetting (CEW)	2.78–3.63	36%	-
[22]	PIFA	Size modification—upper arm	Galinstan/manual-syringe pump	0.698–0.746	-	-
[45]	Slot	Size modification—two slots on patch	EGaIn/manual	2–3.5	70%	-
[32]	Shape	Size modification—shaped pattern patch	Galinstan/manual-syringe	2.2–9.3	-	-
[46]	Double patch	Size modification—two side channels	Galinstan/electrical	14.2–15.1	-	3–3.7 dBi
[47]	Loop	Size modification—flexible tube	Galinstan/manual	0.868–2.45 1.7–1.9	-	-
[23]	Slot	Reactive loading—two separate channels	Galinstan/manual	2.4, 3.5, and 5.8	>2.5	1.2 dBi
[35]	Slot	Reactive loading—five microchannels	Galinstan/manual	1.8–3.1 3.2–5.4	3:1	1.1–3.4 dBi/ 78, 82
[48]	Slot	Reactive loading—open ended channel	Galinstan/manual-syringe	1.85–2.07	11.2%	2.1–4.1 dBi
[43]	Pixelated dipole	Pixels	Galinstan/continuous electrowetting (CEW)	1.68, 1.85, 2.12 and 2.51	13.6–21.6%	±3 dBi/ 70.2–75.4
[49]	Quarter-mode substrate integrated waveguide (QMSIW)	Vias	Galinstan/manual	3.2–4.7	1.45:1	4.6, 5 dBi
[50]	Meander	Floating ground plane	Galinstan/manual-syringe	0.5–3.9	-	1.32–3.12 dBi/ >60

**Table 3:** Summary of polarization reconfigurable antennas using nontoxic conductive liquid.

Ref	Antenna Type	Design Approach/Technology	Liquid Metal/Actuation	Freq. Range (GHz)	Tuning Characteristics/Tuning Ratio	Gain (dBi/dBiC)/Efficiency (%)
[51]	DRA	Glass DRA incorporating liquid metal polarizer	Galinstan/manual	2.4	±45°, 0°	>6 dBi >80
[52]	DRA	DRA incorporating liquid metal and dielectric liquid	Galinstan/manual	2.4	90° to 45°	2–4 dBi >70
[40]	Antipodal Dipole	Antipodal dipole with V-shaped channel	Galinstan/electrical	3	±45°	1.4–2.3 dBi
[53]	Aperture coupled patch antenna	Truncated corner square patch with four triangle cavities of liquid metal	EGaIn/manual-syringe	2.45	LP, LHCP, RHCP	7.24 dBi, 7.25 dBiC, 7.33 dBiC >90
[54]	Truncated-corner patch antenna, Annular slot antenna	Truncated at two orthogonal of square patch, Two discontinuities at 45° and 225° across the slotline	EGaIn/manual–vacuum pump	2.5	LP, CP	-
[45]	Slot antenna	Rectangular patch with two asymmetrical slot and extended patch slot	EGaIn/manual	2.4	LP, RHCP, LHCP	>98

**Table 4:** Summary of antennas with other types of reconfiguration using nontoxic conductive liquids.

Ref	Antenna/Liquid Metal Type/Actuation	Design Approach/Technology	Reconfig. Type	Freq. Range (GHz)	Tuning Characteristics	Gain (dB/dBi)/ Efficiency (%)
[17]	Patch antenna/Galinstan/continuous electrowetting (CEW)	Rectangular patch with inset feed line and LM-filled stub	Gain	5	-	−5.90–4.43 dB
[55]	Helical antenna/EGaIn/manual	Helical antenna with LM-filled turns	Gain	5	-	5–9 dBi
[16]	Two-arm spiral antenna/EGaIn/manual-pump	LM-filled spiral is embedded into silicone elastomer	Directivity	6.9–13.8	*y*-direction (90°) and -*y*-direction (270°)	40–72
[56]	Transmitarray unit cell/Galinstan/manual-micropump	Unit cell with LM-filled split ring structure	Phase	8–10	360° linear phase shift	-
[41]	Dipole antenna/Galinstan/ electrocapillary actuation (ECA)	LM-filled dipole arms are enclosed in polyimide fixtures	Compound (polarization + pattern)	1.579	0°, −45°, 45°, −90°, and 90°	69–97
[18]	Crossed-dipole antenna/EGaIn/ electrochemically controlled capillary (ECC)	Crossed dipole with two pairs of LM-filled dipole arms	Compound (frequency + polarization)	0.8–3	LP (0.8–3 GHz) CP (0.89–1.63 GHz)	41–70
[39]	Helical antenna/Galinstan/manual-peristaltic pump	Helical antenna with LM in flexible polymer tube	Compound (pattern + polarization)	1.575	CP and EP axial beams, LP semidoughnut and axial beams	8.5, 7.6, 1.1, and 5.9 dBi
[57]	Origami antenna/Galinstan/syringe pump	Origami with LM in zig-zag and helical structures	Compound (frequency + polarization + pattern)	3 and 5	LP (3 GHz) to CP (5 GHz) Directional to omnidirectional	-

**Table 5:** Summary of reconfigurable antennas using other conductive and dielectric liquids.

Ref	Antenna/Liquid Type	Design Approach	Reconfig. Type	Freq. Range (GHz)	Tuning Characteristics/Tuning Ratio	Gain (dBi)/ Eff. (%)
[38]	Monopole antenna/Conductive (mercury)	Monopole with capacitive coupling feed line	Frequency	2.5–5	~4:1	1.3–3 dB (monopole) >6 dB (array) 80, 65
[58]	Vivaldi antenna/Conductive (ionized water)	Feed line coupled to slot line	Frequency	3.2 and 4.5	-	87 11 and 10.9 dBi
[59]	Dipole antenna/Dielectric (castor oil and ethyl acetate)	Two bent dipoles with plastic enclosure	Frequency	1.17–1.50 0.9 to 1.44	25% 46%	90–47, 92–82
[60]	Microfluidic microstrip patch/Dielectric (DI water)	Inserting liquid tubes between the patch and the ground plane	Frequency	1.391–1.861	-	>6.7 dBi 68.8–86.3
[61]	Dielectric resonator antenna (DRA)/Dielectric (ethyl acetate)	Container with left and right rectangular sections	Polarization	2.4	LHCP (16.7%), RHCP (16.3%)	>2 dBiC >70
[62]	Dielectric resonator antenna (DRA)/Dielectric (ethyl acetate)	DRA with inner and outer sections	Pattern	3.75–5.37	HEM_11δ_ mode (outer section empty) TM_01δ_ (outer section full)	>3 dBi >80, 50–63
